# Lung transplantation: how we do it

**DOI:** 10.1007/s12055-021-01218-w

**Published:** 2021-09-18

**Authors:** John Santosh Murala, Hashim Muhammad Hanif, Matthias Peltz, Sreekanth Reddy Cheruku, Lynn Custer Huffman, Amy Elizabeth Hackmann, Michael Erik Jessen, William Steves Ring, Michael Alton Wait

**Affiliations:** 1 Department of Cardiovascular and Thoracic Surgery, University of Texas Southwestern (UTSW) Medical Center, 5959 Harry Hines Blvd., 10th Floor, Suite HP10.110, Dallas, TX 75390 USA; 2 Department of Anesthesiology and Pain Management, University of Texas Southwestern (UTSW) Medical Center, Dallas, TX USA

**Keywords:** End-stage pulmonary disease, Operative technique, Lung transplantation

## Abstract

Lung transplantation is considered the gold standard for patients with chronic end-stage pulmonary disease. However, due to the complexity of management and relatively lower median survival as compared to other solid organs, many programs across the world have been slow to adopt the same. In our institution, we started lung transplantation in September 1990. And since then, we performed close to 900 lung transplantations. Here, we describe in detail the operative steps adopted in our institution for a successful lung transplantation. There have been very few variations over the years. We believe that having a standardized technique is one of the important features for success of a lung transplant program.

## Introduction

Lung transplantation (LT) is considered the gold standard for patients with chronic end-stage pulmonary disease. Joel Cooper from the University of Toronto performed the first isolated lung transplant with long-term survival, a single right lung transplant for interstitial pulmonary fibrosis (IPF) in 1983, the patient survived for 6.5 years [1]. In 1987, Alec Patterson from the same institution described the first enbloc double LT in a patient with Alpha-1 Anti-trypsin deficiency [2]. Subsequently, Pasque described the sequential double LT which is now the common practice world over [3]. Since then, thousands of LTs have been performed. However, due to the complexity of management and relatively lower median survival as compared to other solid organs, relatively few programs care for this challenging patient population.

We started LT program at our center in September 1990. Between September 1990 and February 2021, we have performed a total of 887 lung transplants including 24 multi-organ transplants (10 heart–lung, 7 lung-kidney, 7 lung-liver), and 12 re-operative lung transplants for chronic lung allograft dysfunction (CLAD). A total of 670 bilateral lung, 121 right, and 96 left single lung transplants have been performed. Recipient ages ranged from 14 to 79 years (median 58). Indications for LT have included interstitial lung disease (ILD = 427), chronic obstructive pulmonary disease (COPD = 210), combined pulmonary fibrosis and emphysema (CPFE = 25), septic (cystic fibrosis = 126, bronchiectasis = 13), pulmonary hypertension (PAH=61), CLAD=12, and others (13 pts). Pre-operative extracorporeal membrane oxygenation (ECMO) was used in 28 patients. Ex vivo lung perfusion (EVLP) was used in 16 patients. The overall median survival is 6.6 years. The actuarial survival rates at 30 days, 1 year, 5 years, 10 years, 15 years, and 20 years are 97.2%, 89.4%, 58.3%, 34.4%, 16.2%, and 10.6%, respectively. There are only two survivors beyond 20 years and one beyond 25 years. Since the inception of the program, very few modifications have been made in the surgical technique. Standardizing the technique has been one of the strengths of our program.

There are many papers describing the operative technique [4–10]. Many approaches have also been described which include bilateral antero-axillary thoracotomies and minimally invasive approach. However, the aim of this paper is to describe in detail the steps of a standard bilateral LT being performed at our institution. We have previously published procedural steps for lung procurement [11]. Here, we continue with the steps in the recipient operating room (OR). All of these steps are standardized, and here, we attempt to describe in detail every step of a successful LT.

## Operative technique

Donor lungs were harvested using a combination of antegrade and retrograde cold Perfadex (XVIVO Perfusion-PERFADEX® Plus) with the donor lungs stored in hypothermic static storage inflated to functional residual capacity (FRC) [11].

### Anesthesia

The donor is identified as blood group ABO compatible using source documentation three times. Once the donor is identified as suitable for donation, the recipient is brought in the OR. Prior to induction of anesthesia, many transplant centers place invasive arterial catheters to facilitate hemodynamic monitoring. The placement of pre-induction central venous lines and pulmonary artery (PA) catheters may also be considered in high-risk patients. However, these lines are typically placed after induction of anesthesia. Transesophageal echocardiography (TEE) can be used to monitor hemodynamics and guide extracorporeal interventions, when necessary. Cerebral oximetry is increasingly used to evaluate and optimize end-organ perfusion during the operation, with 60% of worldwide transplant centers reporting its use in all LT procedures [12]. Induction of anesthesia should be performed with medications which are selected in context of patient comorbidities, including right ventricular dysfunction and PAH. A survey of LT centers worldwide reported that Propofol (93%) and Etomidate (65%) were the most common induction agents [13]. We use the same agents.

A left sided double-lumen endotracheal tube is most commonly used for intubation and to facilitate one-lung ventilation. Lung protective ventilation with 4–6 ml/kg tidal volumes on one-lung ventilation while limiting plateau pressures and titrating positive end-expiratory pressure (PEEP) to maintain arterial oxygen saturation > 92% is the standard of care. Permissive hypercapnia while maintaining an arterial pH > 7.2 is recommended and well tolerated in these patients [14]. Maintenance of anesthesia is commonly accomplished using inhalational agents but a meta-analysis of 20 studies, enrolling 850 patients, found no difference in outcomes between inhalational and intravenous maintenance agents in patients undergoing one-lung ventilation [15]. After each donor lung is transplanted and the PA is unclamped, the lung should be ventilated with the lowest possible fraction of inspired oxygen (FiO2) (< 30%) to prevent the development of primary graft dysfunction (PGD).

### Intraoperative immune suppression

The regimen used for immunosuppression varies with transplanting center but it is common practice to use a combination of interleukin 2 receptor antagonists (IL2RAs), anti-proliferative agents, and steroids. A recent review of the International Society for Heart and Lung Transplantation (ISHLT) registry revealed that IL2RAs such as Basiliximab are used most often for induction immunosuppression. Mycophenolate and Tacrolimus are used for maintenance immunosuppression.

Our institutional protocol is to administer methylprednisolone 500 mg IV to all patients undergoing LT. Azathioprine (Imuran) is given orally at a dose of 2 mg/kg pre-operatively as the default routine; if patients are nil per oral, azathioprine can be given intravenously. Some patients with ILD are already receiving Mycophenolate (CellCept) prior to their transplant, and if so, then Mycophenolate (1500–3000 mg orally) is continued in place of Azathioprine. Unstable patients, patients with PAH, or patients who require cardiopulmonary bypass (CPB) or ECMO to facilitate implantation are given Basilixumab (Simulect) as an IL2RA in lieu of pre-op calcineurin inhibitors. Otherwise, the more stable patients who do not require CPB are given calcineurin inhibitor Tacrolimus (Prograf), as a continuous infusion at 30 mcg/kg/h.

### Position and incision

The decision to use CPB is made on a case by case basis. Our default position is to do bilateral LT off pump with CPB on standby.

**Fig. 1:**
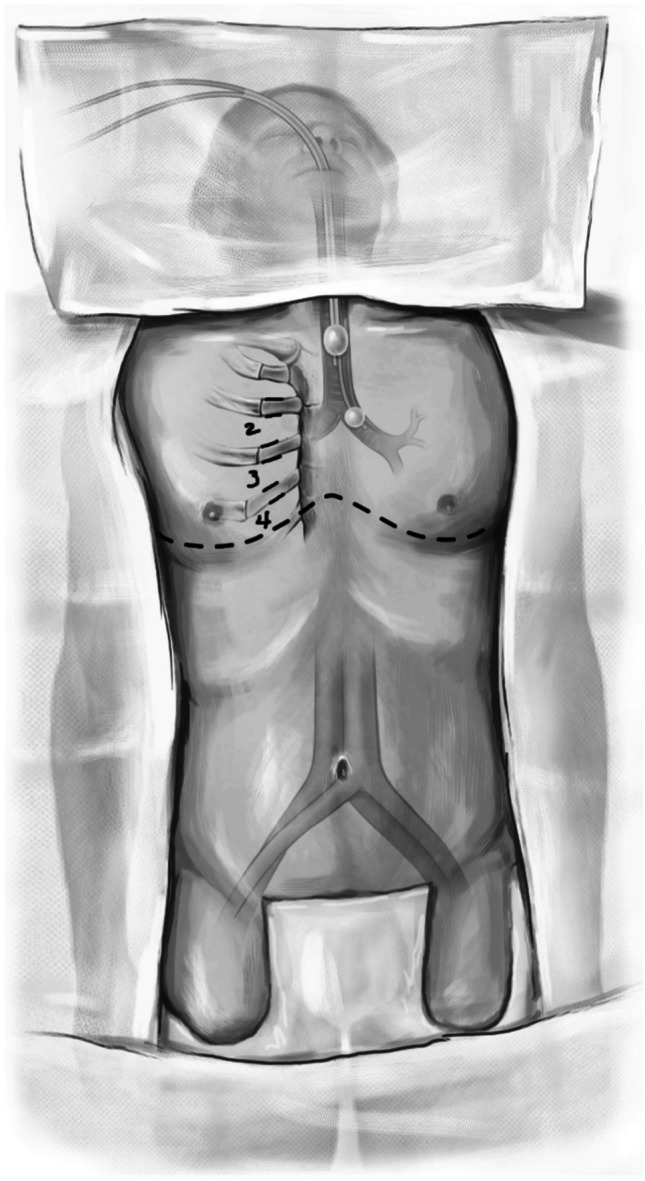
Showing the position and the marking on the chest wall for incision. Both groins are left open for cannulation. Single lung ventilation is planned with left sided double-lumen endotracheal tube

With the patient supine, the neck is extended and the chest is laid flat without any roll at the back. The chest, abdomen, and legs are prepped and draped in a sterile manner, taking care to expose the chest as far laterally as possible. We also keep both groins available for possible cannulation (Fig. 1). Surgical timeout is performed where blood type and donor details are also vocalized.

The instruments needed for LT (off pump) are listed in Table 1.

**Table 1:** Instruments needed

1. Instrumentation
a. Open heart tray
i. Including Satinsky clamps, long needle drivers (or long Castro-viejo, Jacobson needle drivers), sternal wire driver, 2 Forester clamps, long Debakey forceps, long Russian forceps, long Metzenbaum scissors, clip appliers (small, medium, large)
b. Lung tray
i. Clamps needed
Duval clamp × 2, Lee bronchus clamp × 2, bronchus clamp × 1, long Babcock clamp × 2, long Allis clamp × 2
ii. Retractors needed
Weitlaner × 2, Army-Navy × 2, double-ended Richardson, Wilson rib retractor × 2, Tuffier rib retractor × 2, Allison lung retractor × 2 (one big, one small), Harrington retractor × 1
c. Long fine coronary tray
Including fine Castro-viejo needle drivers, long micro-Debakey forceps × 2
d. Sternal saw
Needed only for bilateral LT
2. Suture
a. 4–0 Polypropylene 17 mm half circle taper point needle
Used for bronchial anastomosis (6–7 sutures), pulmonary vein (1 suture)
b. 5–0 polypropylene 13 mm 3/8 circle taper point needle
Used for pulmonary artery (1 suture)
c. 5–0 Polypropylene (repair stitch)
d. 4–0 Polypropylene 26 mm (repair stitch)
e. 2 Polyglactin 54″ (3 sutures)
f. 2 Polyglactin 36″ 4 pack (2 packs)
g. Closing suture
3. Sterile set-up
a. Bone wax (bilateral lung transplants only)
b. 5000u Heparin/500 cc NS for PA and PV
c. 1000 cc Plasmalyte bag × 2
Meant to make slush for the sterile field
d. Staplers
i. Ethicon TA-30 vascular (reloads-30 vascular)
ii. Ethicon Flex-45 (45 vascular)/Echelon 45 (45 vascular)
iii. For bilateral lungs-TA-30 green (single load for dividing bronchus)

**Fig. 2:**
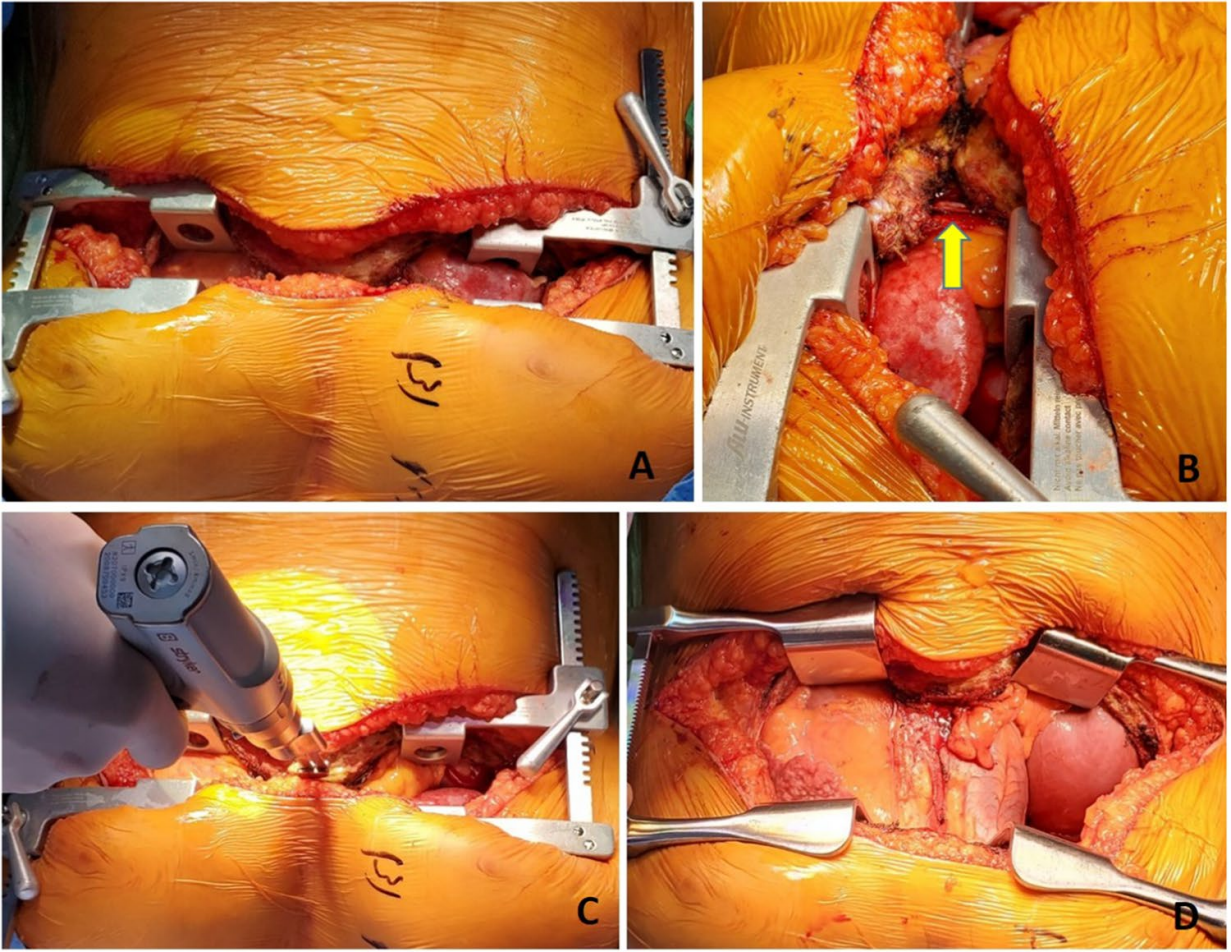
The incision. **A** Bilateral thoracotomies with Wilson retractor as viewed from head end. **B** Right mammary vessels clipped (yellow arrow). **C** Sternotomy performed. **D** Tuffier retractors placed on either side — showing an elevated dome of diaphragm on the right side

The site of the proposed incision (bilateral anterior thoracotomy) is marked. We use a clam shell incision or bilateral antero-lateral thoracotomy (sternal and internal mammary artery [IMA] sparing technique) based on patient characteristics. Often, we need a clam shell incision in patients with ILD who have a shrunken thoracic cavity and a sternal sparing option for COPD patients. A clam shell incision is made and the 4th intercostal space is entered. Initially, we use the Wilson rib retractor to dissect the intercostal space and the mammary vessels (Fig. 2). If sternotomy is needed, the mammary artery and vein are clipped with medium/large clips, divided and the sternum is divided.

The Wilson retractors are removed and two Tuffier retractors are placed as far posterior as possible which often may require some undercutting to facilitate opening the cavity widely. The undersurface of the sternum is dissected. The pericardium is often left unopened if CPB is not required, although some retraction sutures may be placed to retract the unopened pericardium (particularly for left lung implantation). The goal is to keep cardiac retraction to a minimum.

### Pneumonectomy

**Fig. 3:**
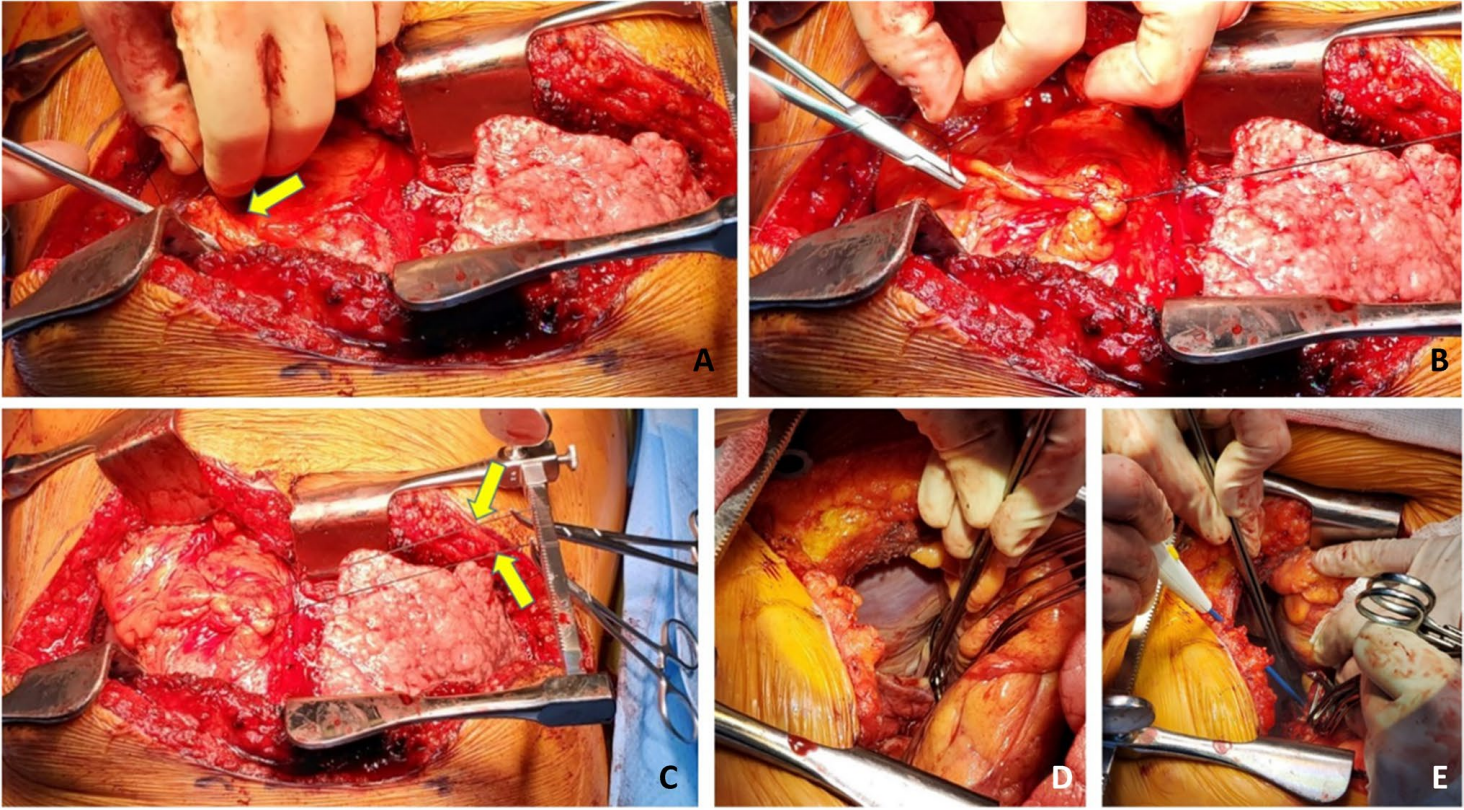
Exposure of left thoracic cavity. **A** Retraction suture placed in the mediastinal fat over the pericardium (yellow marker). **B** Similarly, a second suture is placed. **C** Both the retraction sutures are pulled across the right lung retracting the pericardium and opening up the left thoracic cavity (yellow markers). Also note that the Tuffier retractor is moved closer to the sternum to facilitate wider opening. **D** A lung retractor can be used to retract the left hilum. **E** Duval clamps are used on hilar structures and retracted medially for dissection of hilum. All the above pictures are as viewed from head end

The side of first pneumonectomy is selected based on the perfusion scan (performing the less perfused lung first), ease of dissection, chest cavity condition, etc. It is often easier to start with the right lung first. Once the side is chosen, subsequent steps of the operation are achieved with selective single lung ventilation of the opposite lung. With the lung collapsed, we proceed with the recipient pneumonectomy. Here, we describe the left lung being done first. Often, the left thoracic cavity has limited space and is obliterated with the protruding left ventricle (LV). The hemodynamics may be very labile while doing the left lung implant. We often place a suture in the mediastinal fat over the pericardium or in the pericardium over the LV and use it to retract the heart. The Tuffier retractor on the opposite side is often moved closer to the sternum and opened wide and a lung retractor is used to gently expose the hilar structures. Various maneuvers done to expose the left thoracic cavity are shown in Fig. 3.

**Fig. 4:**
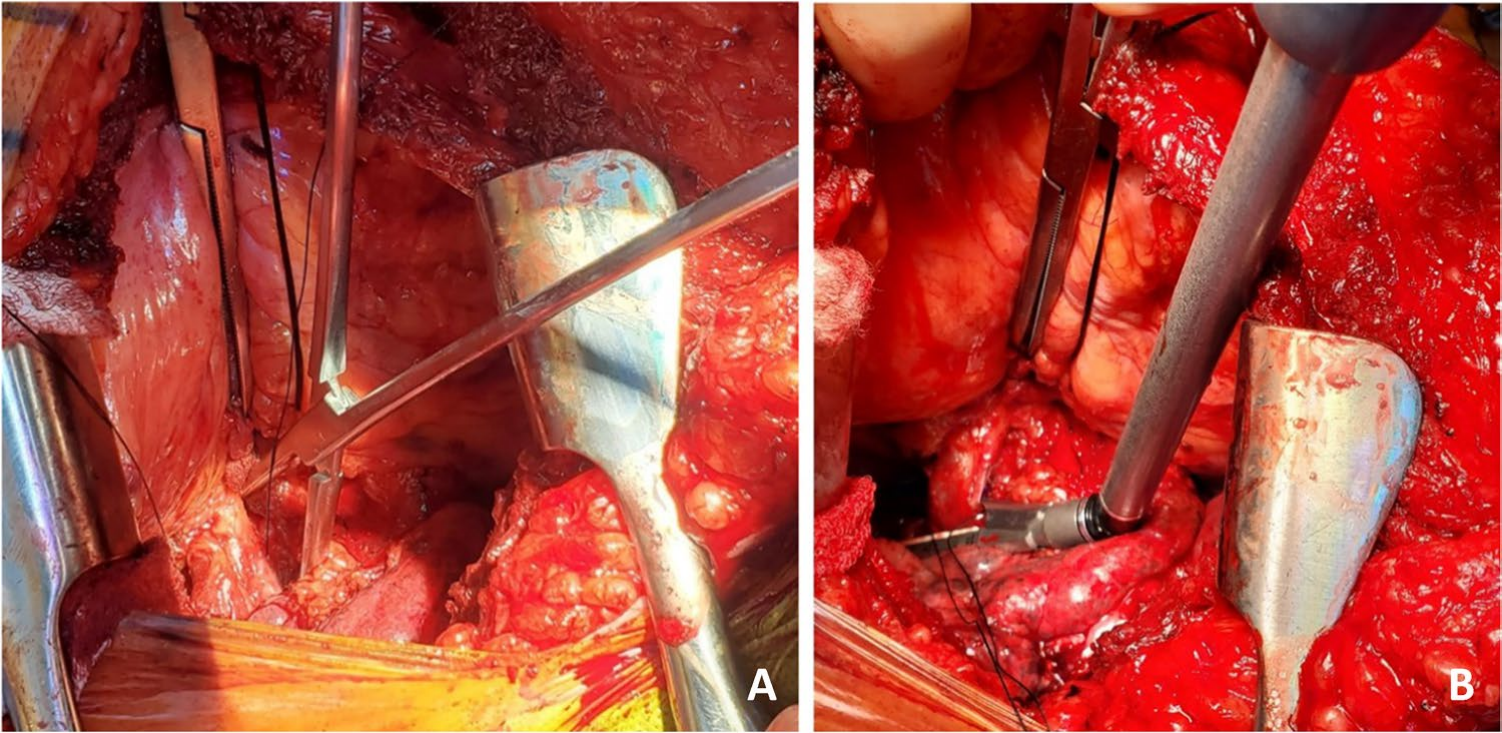
**A** Pulmonary vein dissected and looped with silk. **B** Echelon 45 vascular stapler across the pulmonary vein

With the lung collapsed, dissection is started with the pulmonary veins. Each pulmonary vein is dissected with electrocautery and a right-angled clamp is passed posteriorly. They are then looped with silk suture and are doubly tied. The pulmonary veins are divided between the two ties. Alternatively, a flex 45 stapler (Ethicon ENDOPATH ETS-Flex 45) or ECHELON FLEX 45 Powered Vascular Stapler (Ethicon Echelon Flex GST System) can also be used (Fig. 4). The inferior pulmonary vein is identified by lifting the lower lobe with a Duval clamp and dividing the inferior pulmonary ligament using LigaSure (Medtronic LigaSure™ Maryland Jaw). Note here that often the inferior pulmonary vein is stapled across vertically in order to keep the orientation of the left atrial (LA) cuff.

**Fig. 5:**
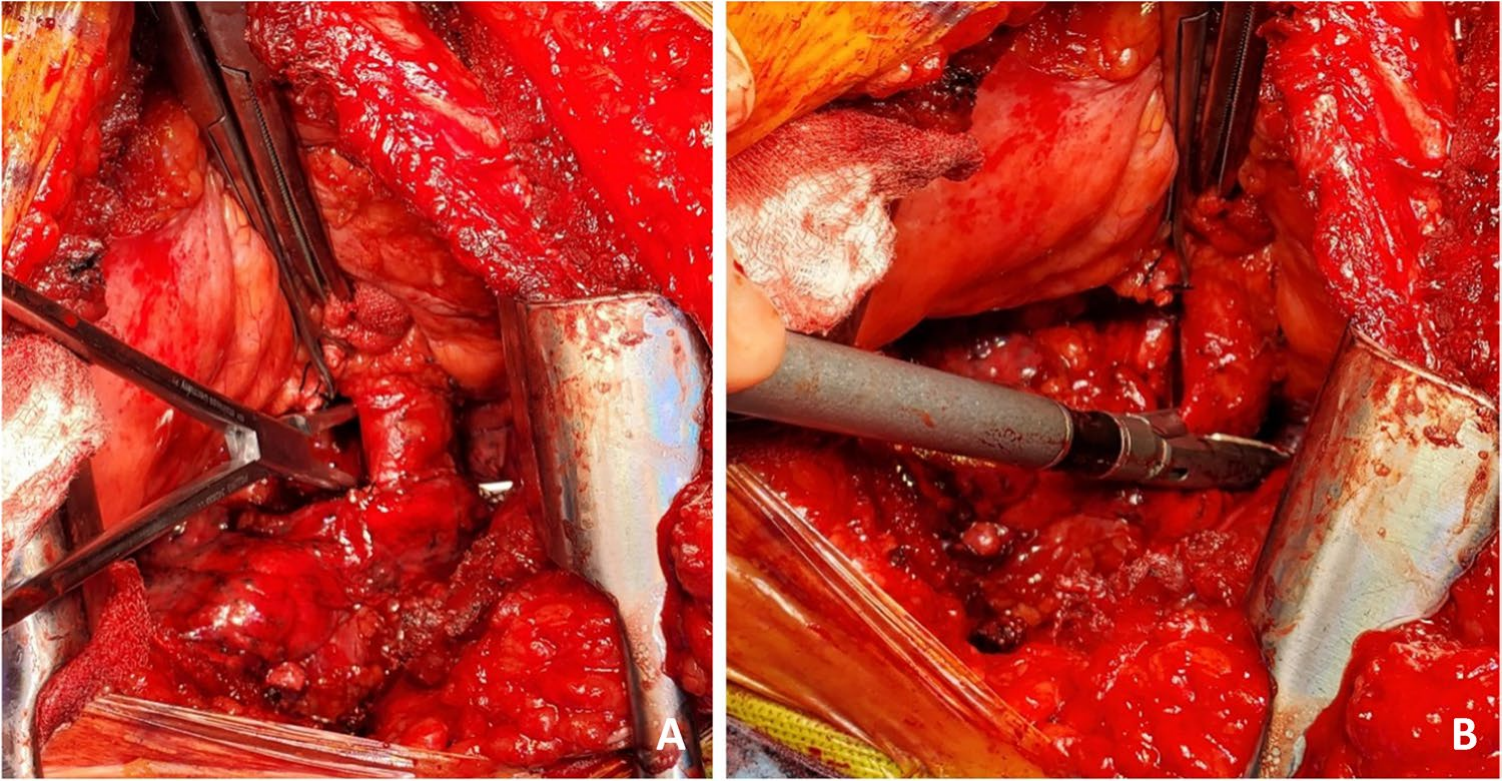
**A** Left pulmonary artery dissected. **B** Echelon 45 vascular stapler across the pulmonary artery

Once the pulmonary veins are divided, the PA is dissected using diathermy, blunt, and sharp dissection. A right-angled clamp is passed around the PA followed by a bronchus clamp. The superior or truncus anterior branch is looped with silk and divided using the Echelon 45 vascular stapler (Ethicon Echelon Flex GST System) once (Fig. 5) or the TX 30 vascular stapler (Ethicon Proximate TX Reloadable Linear Stapler) twice (this is discussed in more detail in right pneumonectomy section). Then, the intermedius is dissected and looped with silk and divided with ECHELON FLEX 45 Powered Vascular Stapler or TA 30 vascular stapler.

**Fig. 6:**
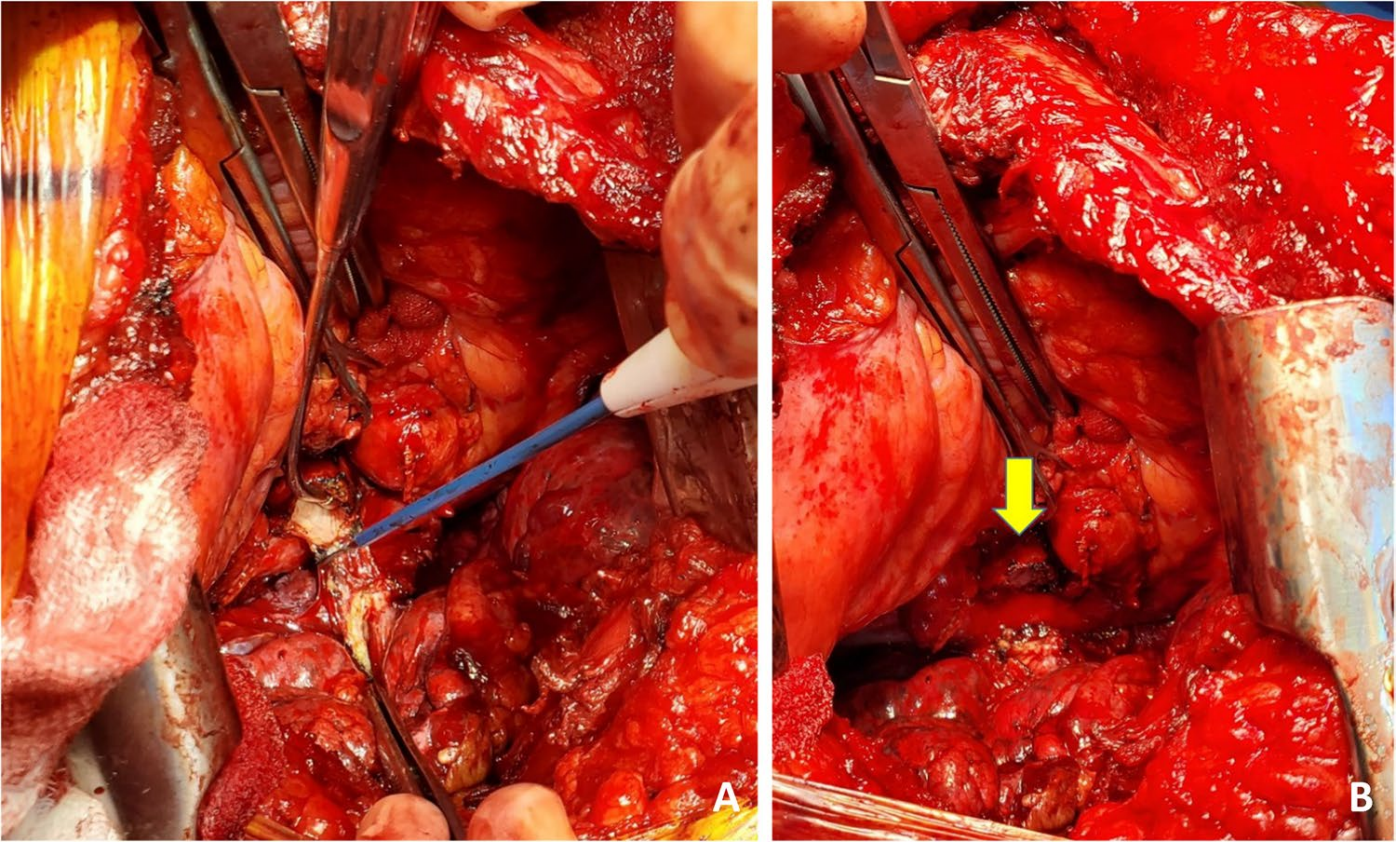
**A** Left bronchus being divided. The membranous portion is divided using diathermy. **B** Divided bronchus (yellow marker)

Next, the bronchus is exposed. The oxygen supply is disconnected from the endotracheal tube (to prevent diathermy flash fire, which can occur if high concentrated oxygen comes in contact with diathermy) and the bronchus is cut open and divided with a 15-blade scalpel (Fig. 6). Thus, the left pneumonectomy is completed.

### Preparing the hilum

**Fig. 7:**
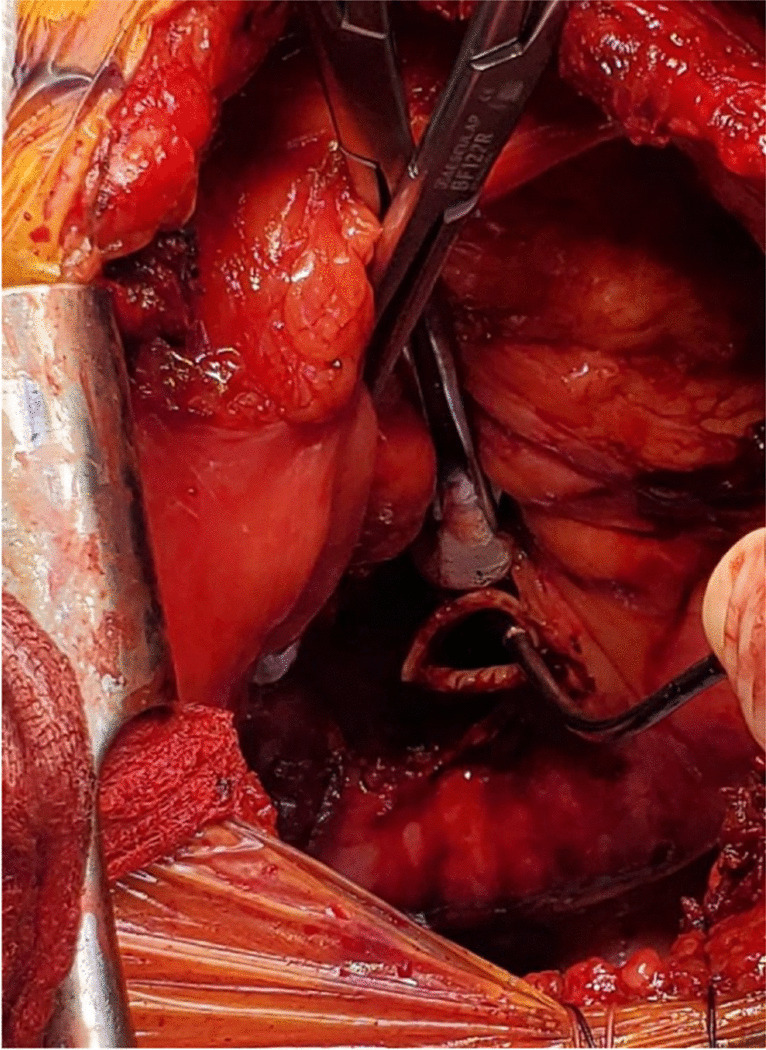
The hilar structures are retracted using sponge sticks exposing the bronchus. The bronchus is retracted using a metal Yankauer suction tip and surrounding tissues are dissected

The next important part of the operation involves dissection of the hilar structures. The heart is retracted with silk sutures on the pericardium. The assistant uses two sponge sticks with sponge balls at the end to retract the hilum with minimal hemodynamic disturbance or use the lung retractor. The assistant can also use his hand with strategically curving fingers to retract the pulmonary veins and the artery in the hilum while the surgeon is dissecting. Often, this needs to be done in stages with intermittent relaxation of heart to help with hemodynamics. The bronchus stump is prepared first (the reverse order to the dissection above). A metal Yankauer sucker tip is used to suck and retract the open bronchus medially while the surrounding tissues are dissected (Fig. 7).

Dissection in this area needs to be meticulous and has to be done carefully. Often, there are many enlarged bronchial vessels which will bleed and are difficult to control. The goal is to preserve most of the soft tissue around the bronchus and not to de-vascularize the stump.

**Fig. 8:**
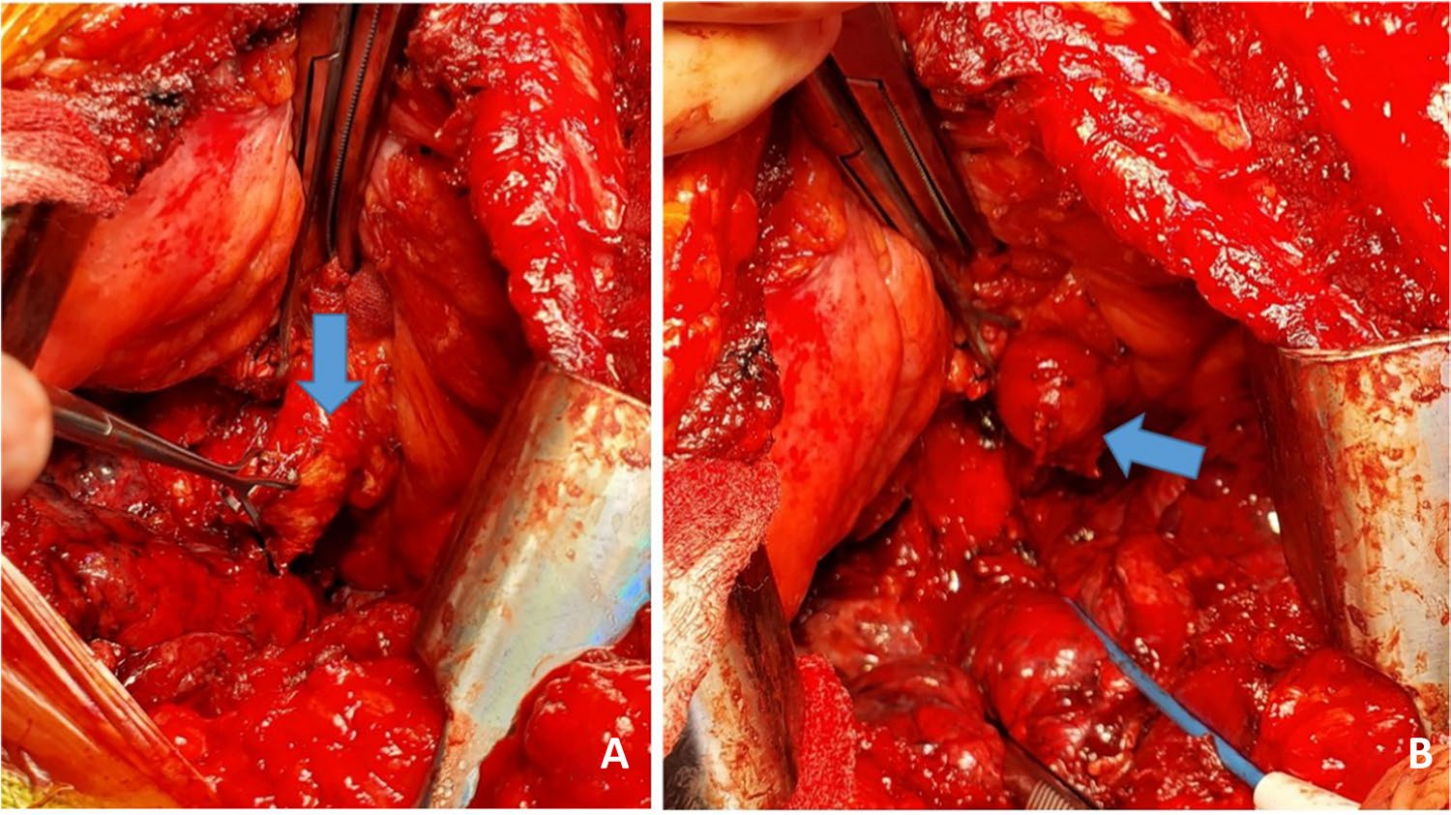
**A** Babcock forceps on the pulmonary stump (blue marker) and surrounding tissues dissected. **B** Long pulmonary artery pedicle (blue marker)

Next, the PA is dissected. The stapled edge is lifted using a Babcock forceps/Duval clamp and initially pulled towards the surgeon (Fig. 8A) and cleared anteriorly and later is retracted medially by the assistant enabling the posterior dissection of the PA. The key is to create a long pedicle (Fig. 8B).

**Fig. 9:**
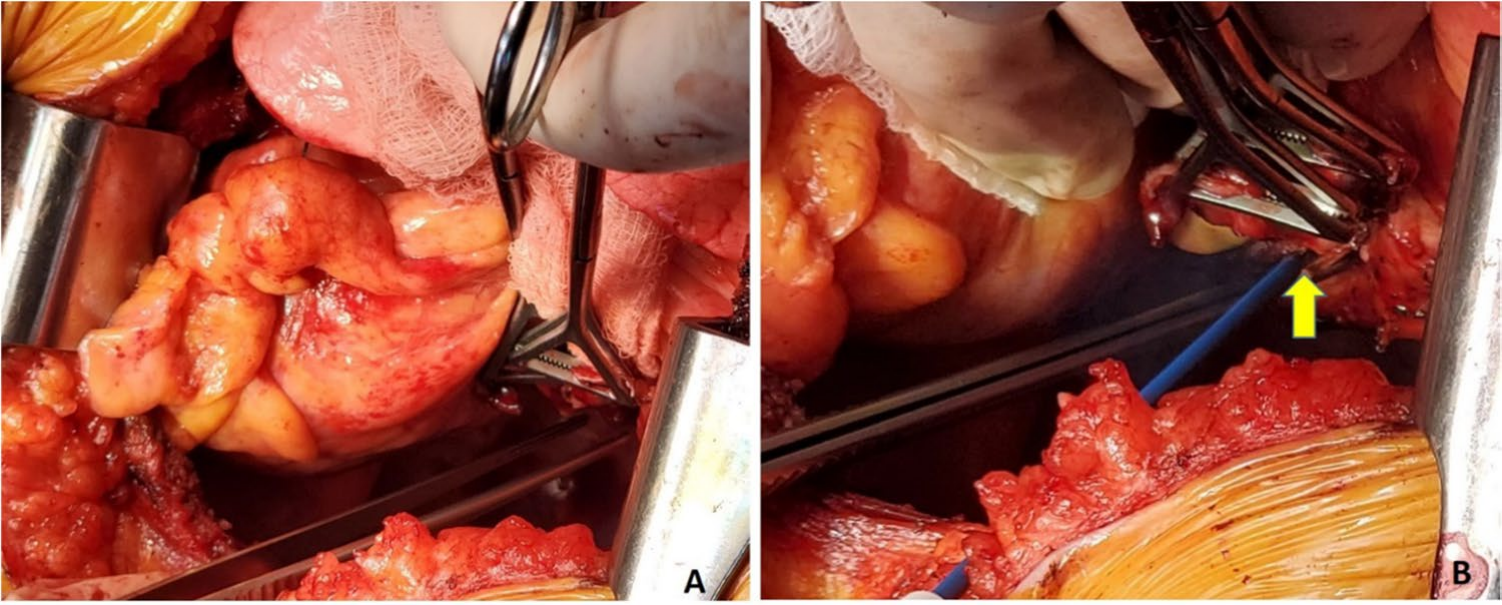
Pulmonary vein cuff being prepared. There is limited space. **A** The pulmonary veins being held by a Duvall clamps and retracted towards the assistant to dissect the intra-pericardial portion. **B** The intra-pericardial portion of the left atrial cuff being dissected (yellow marker)

Finally, the pulmonary veins are dissected. Two Babcock forceps/Duval clamps are used to pull the pulmonary veins first towards the surgeon (Fig. 9). The pericardium is entered anteriorly and the assistant can use right-angled clamp to help dissect the intra-pericardial portion of the pulmonary veins. The pulmonary veins are all mobilized as a LA cuff. This is the difficult portion of the dissection and care must be taken to avoid injury to the left atrium and the pulmonary veins at the base. These maneuvers also carry significant risk of hemodynamic disturbance. Retraction by the assistant is key and often the retraction needs to be released to enable hemodynamic recovery.

**Fig. 10:**
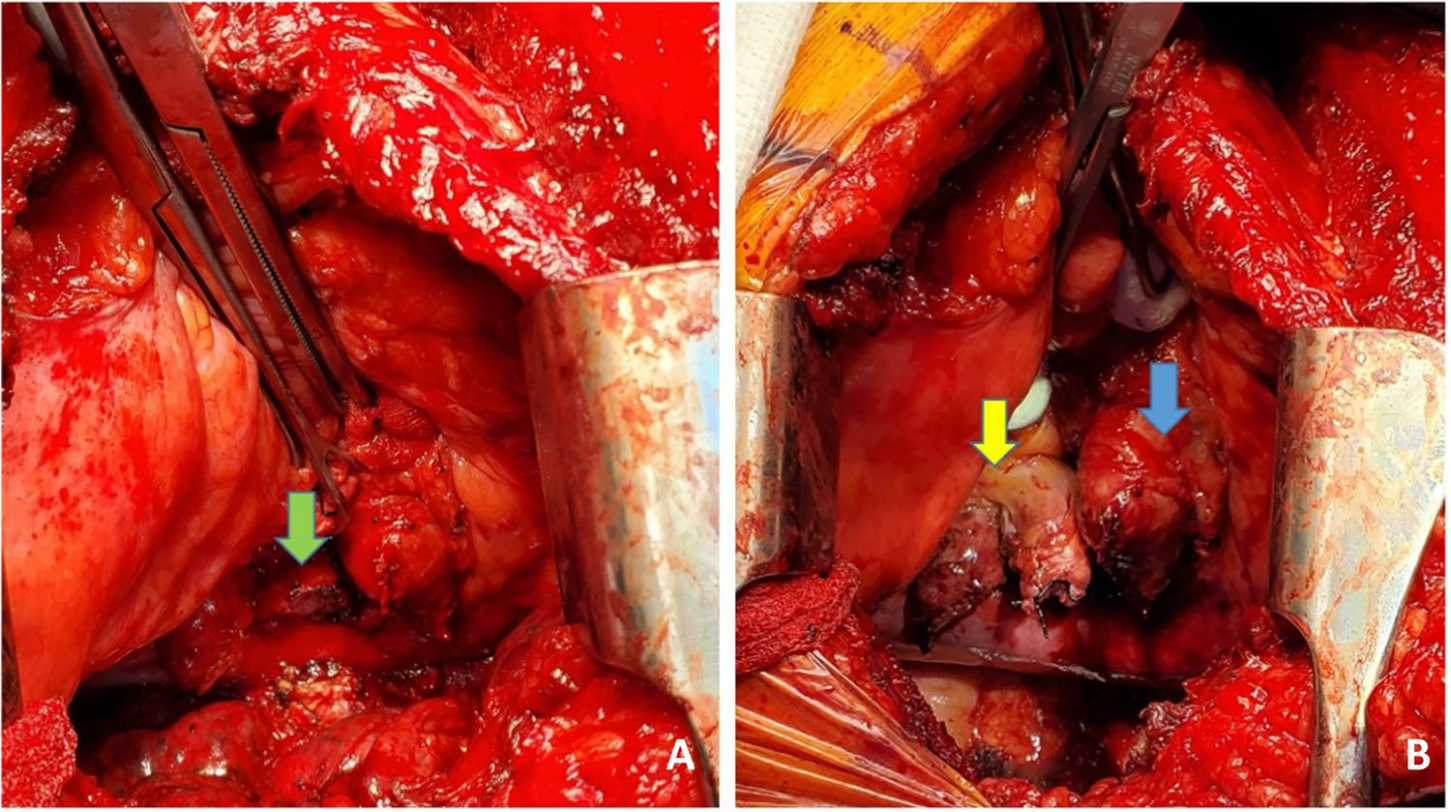
The hilar structures all dissected. The key being long pedicles. **A** Prepared bronchial stump (green marker), **B** left atrial cuff (yellow marker), pulmonary artery cuff (blue marker)

**Fig. 11:**
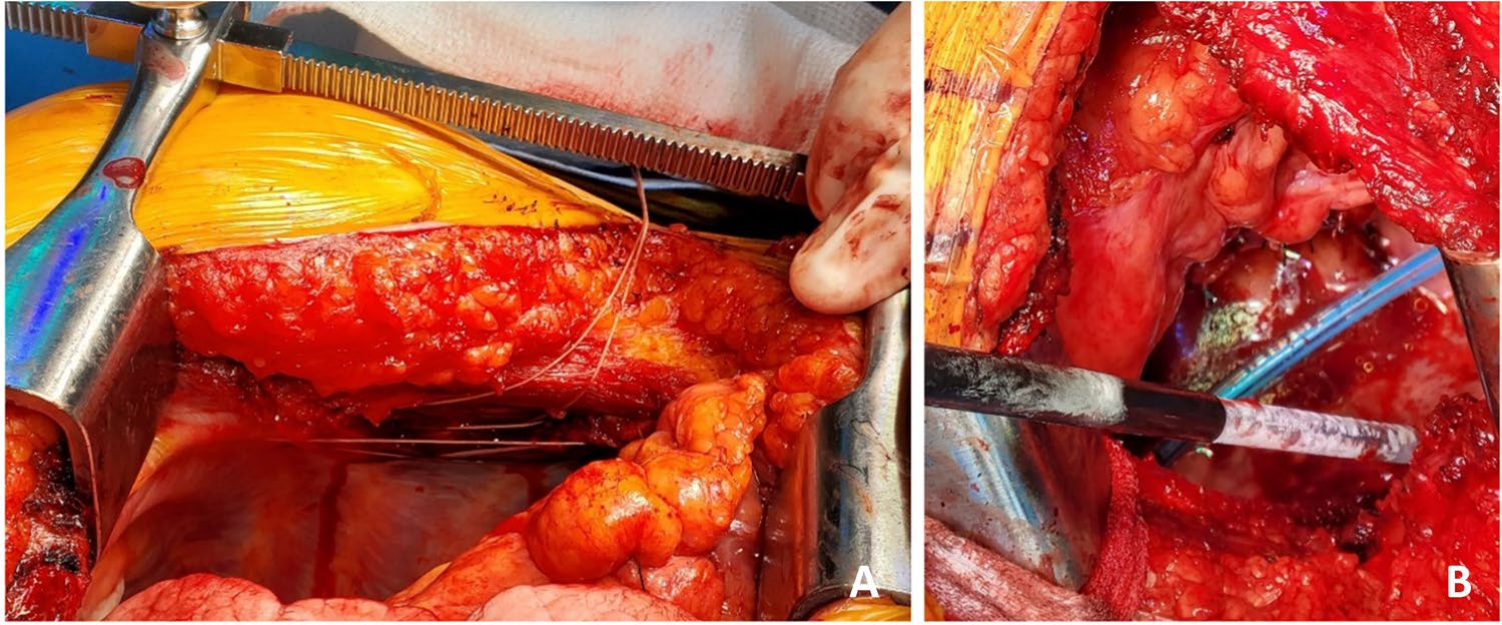
**A** The lateral most para-costal sutures are placed before the implantation. **B** Cryo-analgesia to intercostal space

Once the hilum is dissected (Fig. 10), hemostasis is secured and the rest of the pleural cavity is checked for any bleeding. Laparotomy pads are used to make sure the bed and pleural surface are hemostatic. Here, it is important to place the most lateral para-costal stitches since exposure is best at this stage when the pneumonectomy is completed and prior to the new lung being implanted. We use No.2 absorbable, synthetic, braided Polyglactin suture (54″) as a figure of 8 suture and the ends are placed on a hemostat and placed laterally out of the field. Similarly, a second suture (No.2 Polyglactin 36″) is used with a hemostat at the end (Fig. 11A).

We have been recently trying to facilitate narcotic free analgesia to prevent and treat acute post-thoracotomy pain [Internal Classification of Diseases ICD-10 G89.12]. Here, the 3rd–6th intercostal nerves are treated with cryo-analgesia by placing the AtriCure cryoICE cryoSPHERE Nitrous Oxide (N_2_O) system (Atricure cryoICE® cryoSPHERE™ cryoablation probe). This is performed bilaterally. The probe is placed on the individual T3 through T6 intercostal bundles causing rapid heat extraction to result in Wallerian degeneration (Fig. 11B). Total time required for multilevel intercostal nerve cryo-ablation is about 10 min on each side following pneumonectomy but prior to implantation of the donor lung.

### Back table preparation

When the donor lungs are brought into the OR, an ABO verification is once again confirmed using source documentation.

**Fig. 12:**
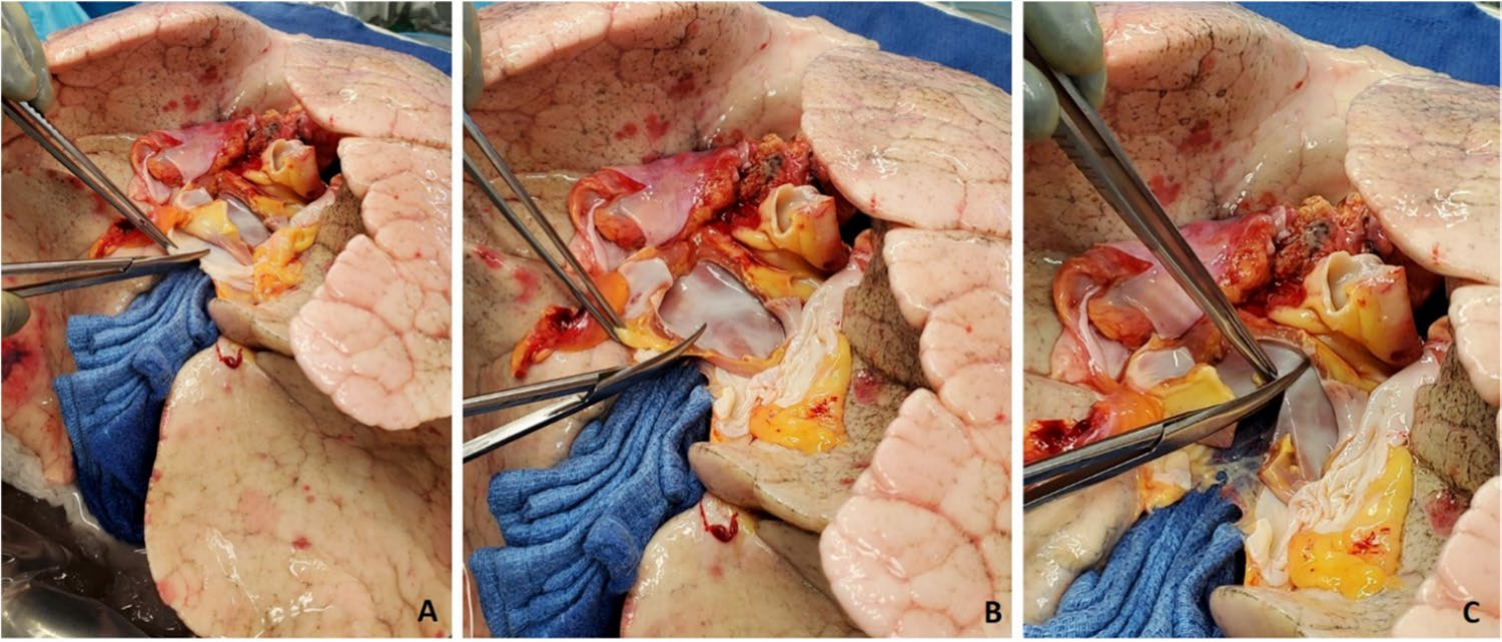
**A** Pericardium being divided. **B** Left atrial cuff divided in the middle. **C** Completing division of LA cuff (note the offset with the pulmonary artery bifurcation)

Next, the back table preparation of the lung block is performed. The lungs are removed from the transport container in a sterile manner and placed on an iced laparotomy pad on the back table. The dissection is made easier if three folded towels are placed behind the hilum to wedge the allograft. The excess pericardium is excised. Pericardium and left atrium are divided in the midline separating the right and left vein cuffs. The midline of the PA bifurcation is slightly offset from the midline of the LA cuff. Care needs to be taken after completing the LA cuff division to prevent damage to the right PA. The soft tissues are further dissected exposing the PA bifurcation (Fig. 12).

**Fig. 13:**
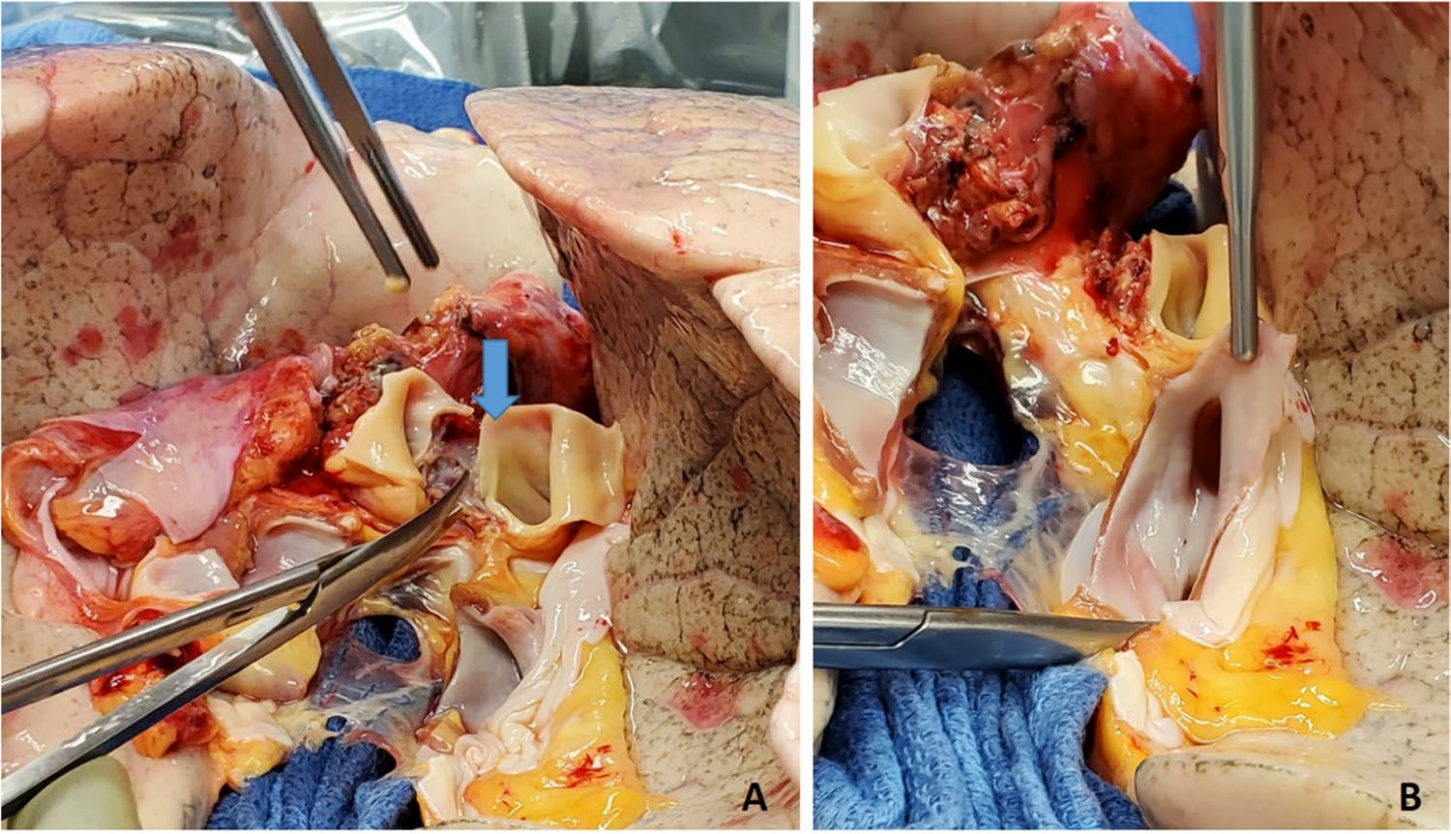
**A** Showing the division of PA to the right side of the raphe (blue marker). Note the offset of pulmonary bifurcation from the midline of LA. **B** Left pulmonary venous cuff

The PA is divided at the bifurcation (Fig. 13). Often, we “cheat” to be on the right side of the raphe as to preserve the length of the left PA (LPA). Both the pulmonary arteries are mobilized into each respective hilum.

**Fig. 14:**
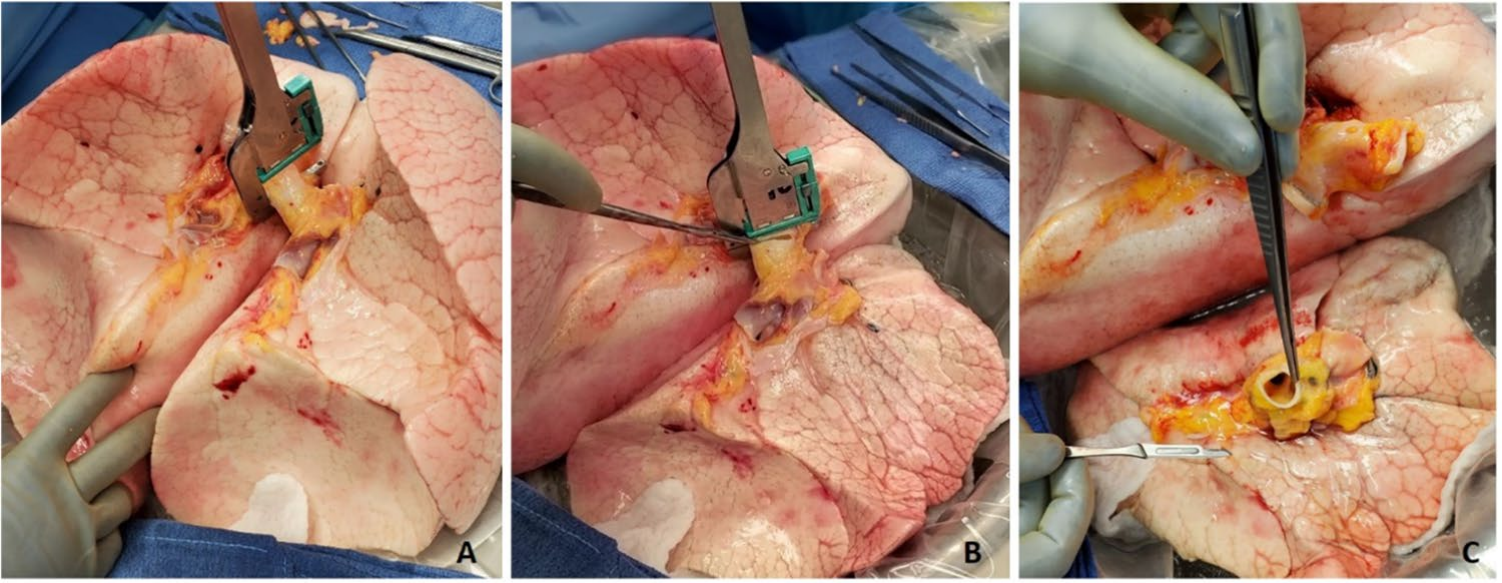
**A** Left bronchus stapled with TA 30 vascular staple. **B** Bronchus divided and the lung is deflated. **C** Bronchus fashioned

The soft tissues are dissected to expose the left bronchus at the carina. A TX-30 green stapler (TX-30 green stapler; Ethicon PROXIMATE TX Reloadable Linear Stapler) is placed across the proximal left bronchus and fired (Fig. 14A). Depending on which lung is implanted first, the bronchus is cut proximally or distal to the stapled line. The lung to be implanted collapses (Fig. 14B) and the other lung is wrapped in moist laparotomy pad and is stored in the semi-inflated stage in ice cold saline bath. A bronchial culture swab is obtained from the donor and recipient bronchus and passed off the field.

The PA is trimmed. Excess LA and pericardial tissue is trimmed with minimal dissection of the bronchus to preserve blood supply. The bronchus is transected and trimmed with a #15 blade to about 2 rings from the upper lobe take-off on right side and 2 tracheal rings proximal to the sub-segmental carina on left (Fig. 14C).

The donor lung is now brought to the surgical field.

Similarly, when the second lung is ready for implantation, the bronchus is divided proximal to the staple line and lung is deflated. The back table preparation is completed in a similar way described above. The bronchus is dissected out and transected with a #15 blade. The PA is also prepared for the anastomosis. The pulmonary vein cuff is also prepared.

### Left lung anastomosis

**Fig. 15:**
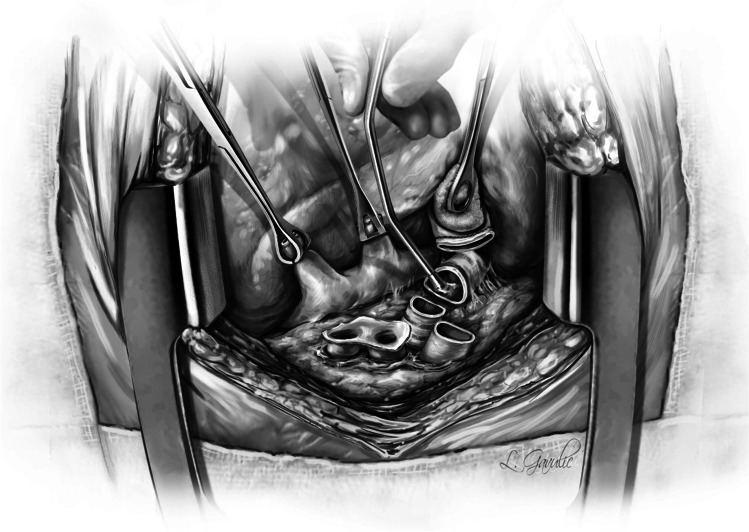
Hilar structures shown. The pulmonary veins are retracted using Babcock forceps and pulmonary artery using a sponge stick. The bronchus is sucked and is ready for the anastomosis. The donor lung is placed in the costo-mediastinal sulcus

**Fig. 16:**
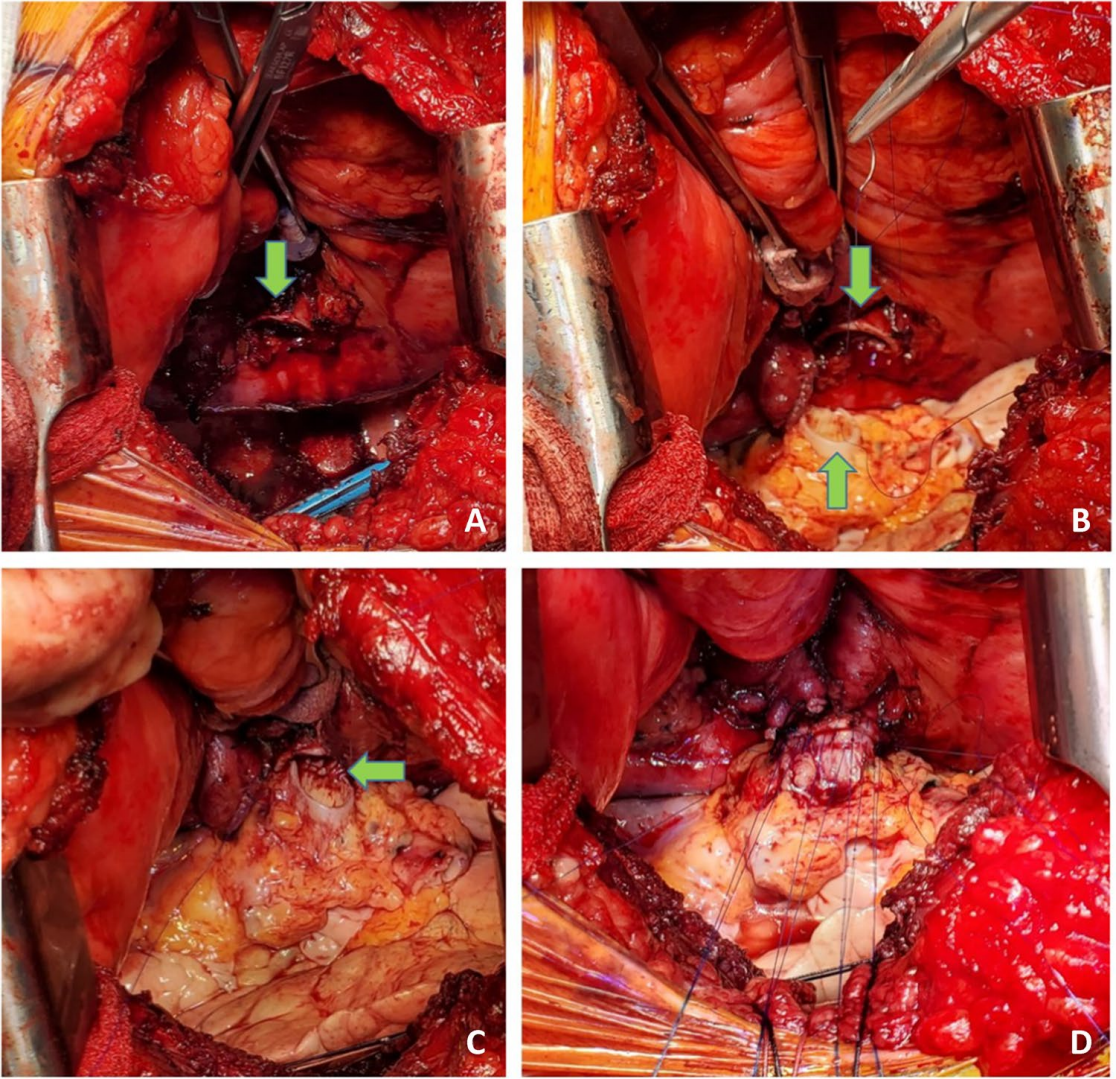
**A** Left bronchial stump is exposed (green marker). **B** Donor and recipient bronchus are aligned (both green markers). **C** The membranous portion sutured with continuous suture (green marker). **D** Multiple figure of 8 sutures placed and are held taut before tying them

**Fig. 17:**
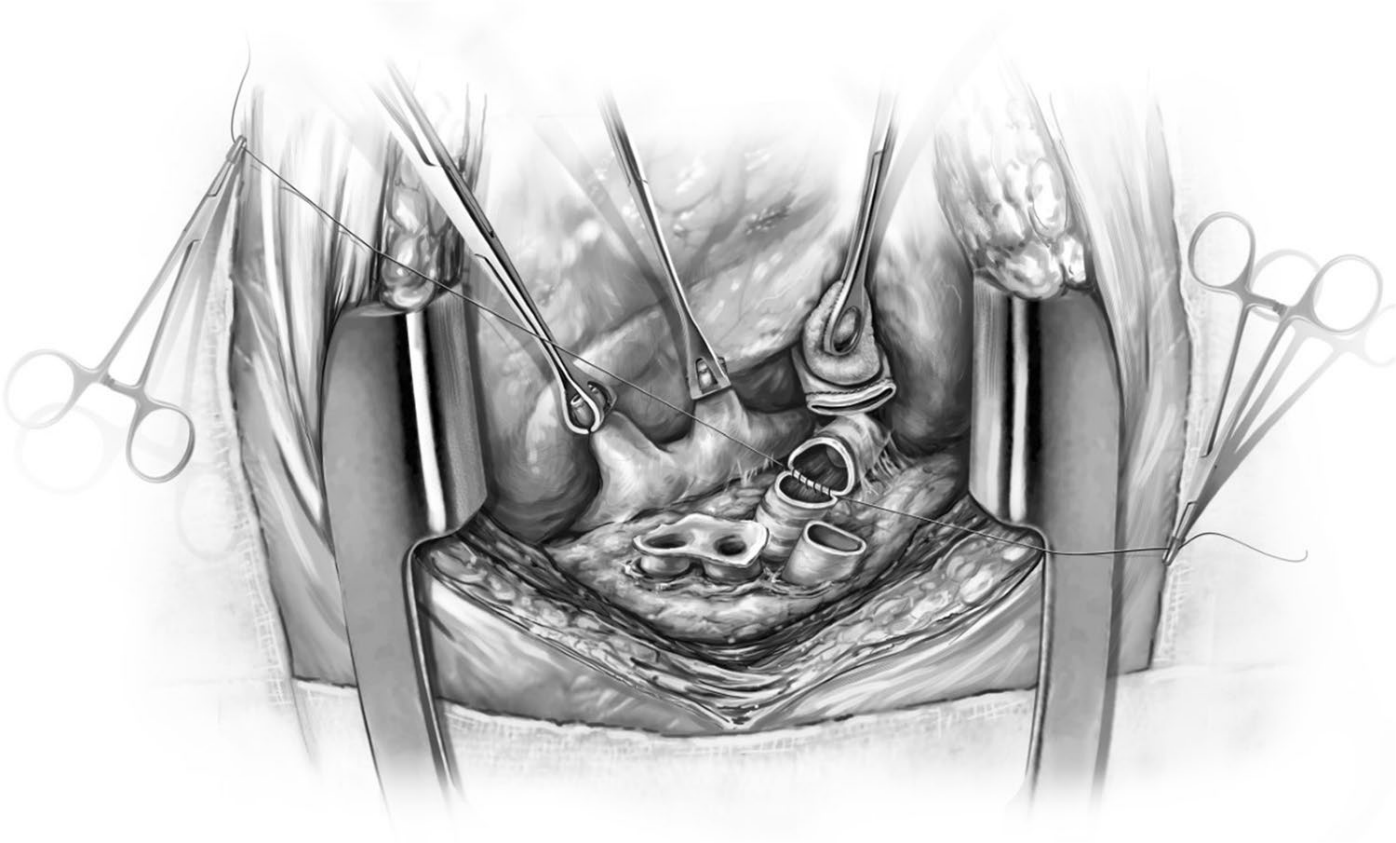
The membranous portion of the bronchial anastomosis is completed. Note that both ends are fixed to the drape to maintain tension

The left donor lung is placed into the costo-mediastinal sulcus (Figs. 15, 16A and B) and a timer is started to note the warm implant time. Please note that the warm ischemic time starts from the time the donor lung is placed in the recipient’s chest. The back bench is all done in cold and the lung is still kept in cold up until it is ready for implantation. The first anastomosis is the bronchial anastomosis. A 4–0 Polypropylene with half circle taper point needle is used for continuous suture of the membranous portion of the bronchus. The first stich is backhand out-in on recipient bronchus at the membrano-cartilaginous junction and in–out at the same junction in the donor bronchus. Then, forehand running sutures are completed from donor to recipient and membranous portion anastomosis is completed. After completion of the last stitch, the suture line is tightened by gentle traction on either end and are fixed to the drape with rubber shod clamps to keep the membranous anastomosis taut (Figs. 16C and 17).

**Fig. 18:**
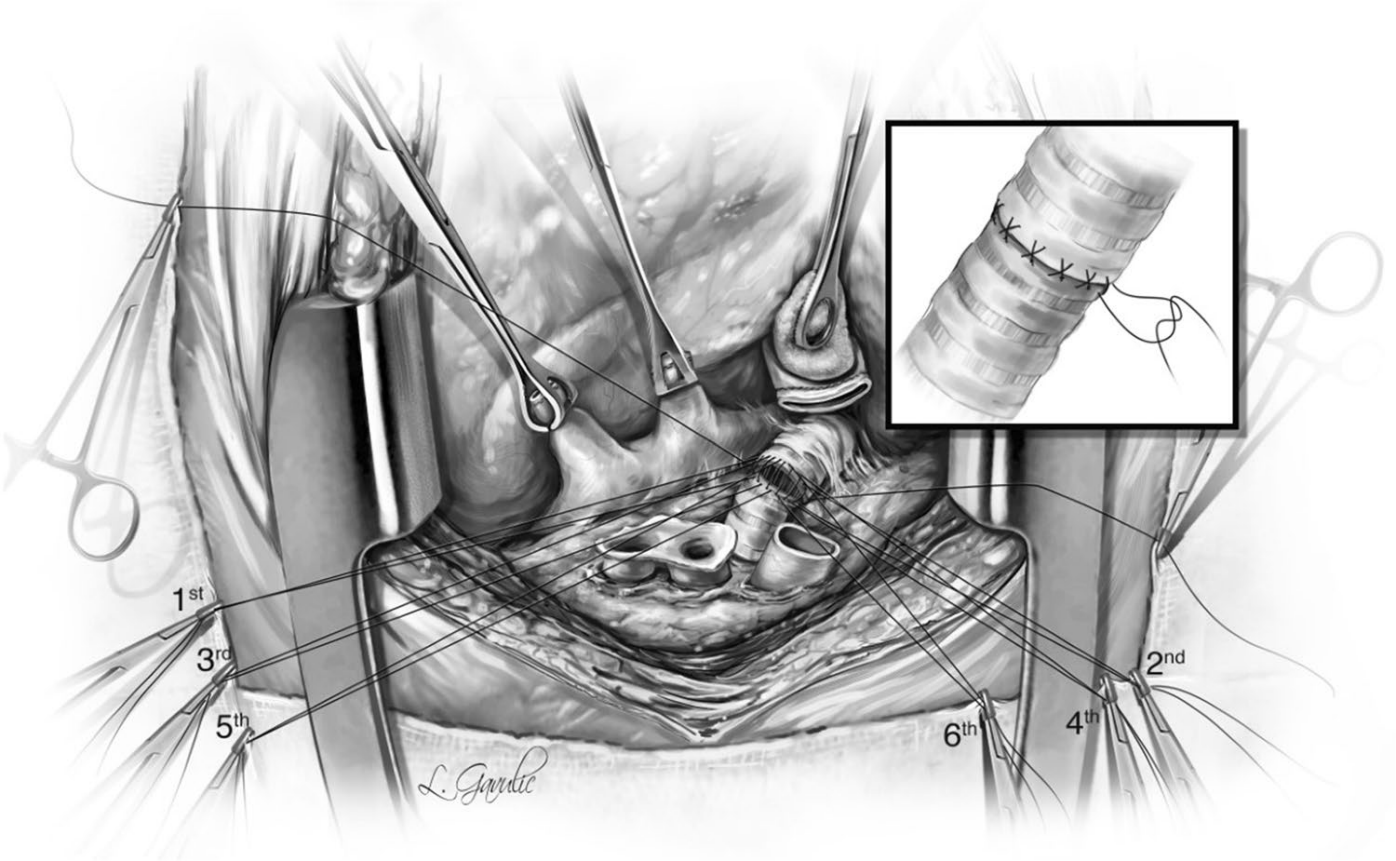
Interrupted figure of “8” sutures are placed in the cartilaginous portion. This is a rough representation of how we place figure of “8” sutures sequentially from either ends and move towards center. Inset when the figure of “8” sutures are tied, tension is applied on all sutures. On either ends, one end of the figure of “8” suture is tied to the end of the continuous suture

Next, five to six interrupted figure-of-eight sutures using 4–0 Polypropylene with 17 mm half circle taper point needle are taken on the cartilaginous bronchus. These are forehand bites starting from each end of membranous-cartilaginous junction and moving to the middle of the cartilaginous portion. Both the ends of each suture are sequentially fixed to drape with rubber shod clamps (Fig. 18). At the completion of all the sutures, tension is applied on all sutures and the bronchial ends are approximated (Fig. 16D). Depending on the size of the donor and recipient bronchus (sometimes D- > R, R- > D, D = R), we attempt to intussuscept into each other. Sometimes, both are absolutely equal and this maneuver may not be possible and they need to be oriented end to end.

**Fig. 19:**
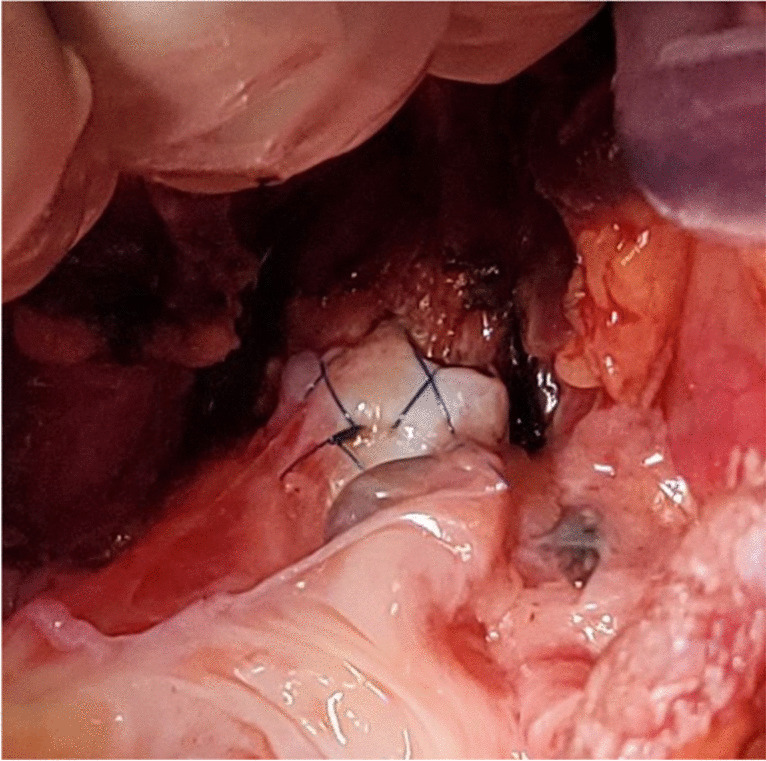
Completed bronchial anastomosis. Note a perfectly placed sutures form “X” on the anastomosis. Here, the recipient bronchus has been intussuscepted into the donor bronchus

The preferred strategy is to take smaller bites on the donor and more on the recipient to telescope donor into recipient. Occasionally, both are equal sized and it is appropriate to align them end to end. Keeping all the sutures under tension, tying is started with the ‘central’ figure -of-eight suture and alternating on either side till the last figure-of-eight suture is tied. On each side, one end of the last suture is left intact after tying and is used to tie the end of the continuous suture of the membranous suture. This completes the bronchial anastomosis (Fig. 19) and at this stage, the anesthesiologist is asked to perform flexible bronchoscopy to assess the anastomosis and assure there is no anastomotic narrowing.

**Fig. 20:**
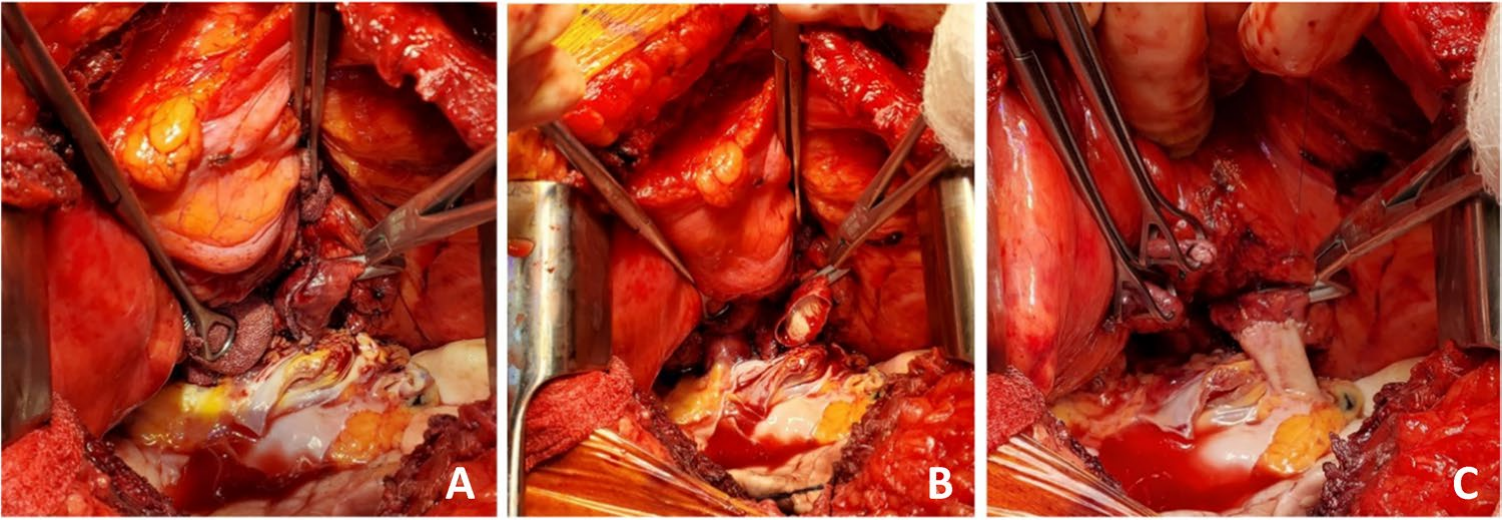
Set up for a PA anastomosis. **A** The pulmonary artery is clamped with a Lee bronchus clamp, wrapped with opened gauze, and fixed to the drape. **B** The stapled edge has been opened and the PA is trimmed. **C** Completed PA anastomosis

**Fig. 21:**
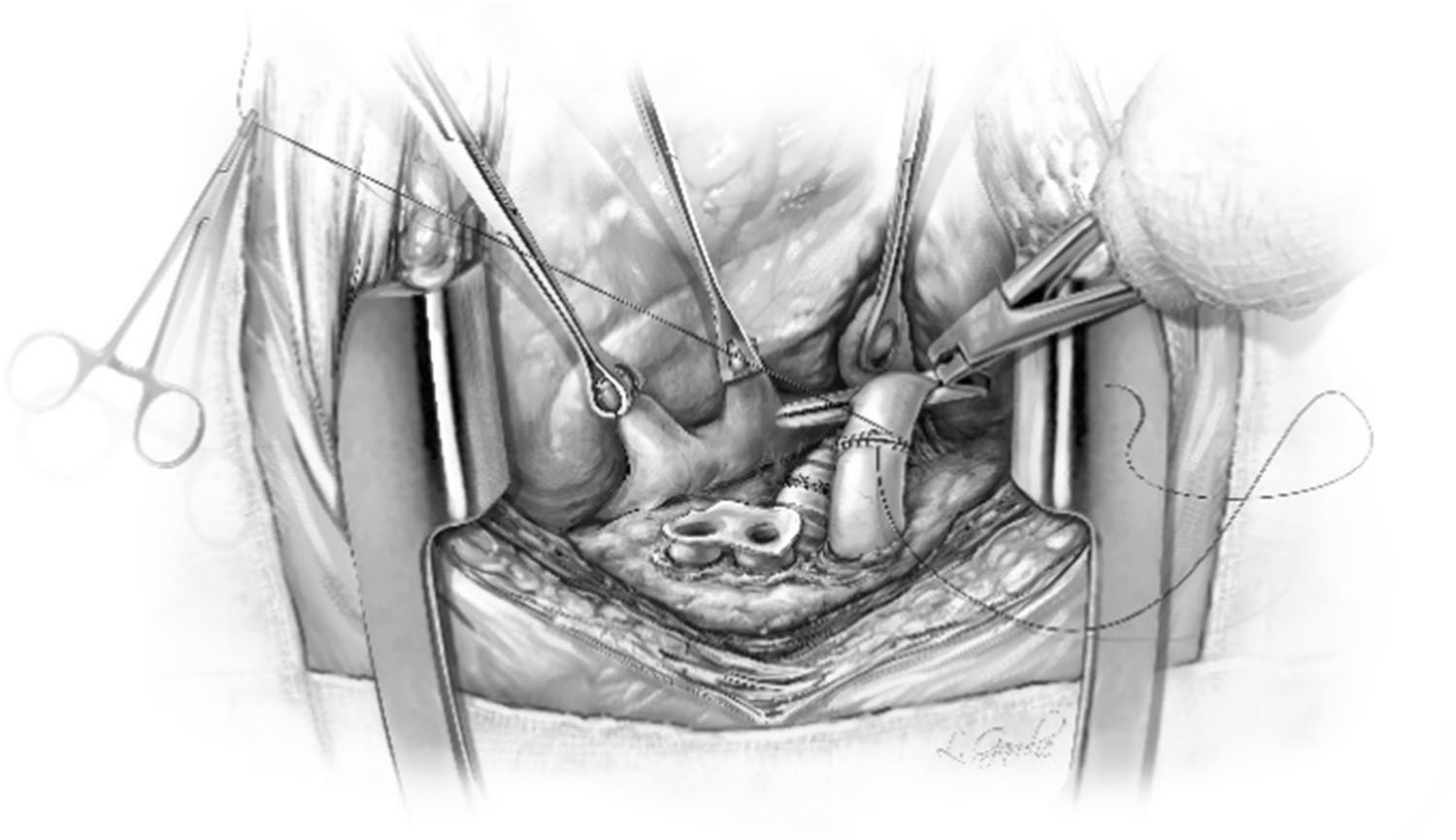
Anastomosis of PA completed and the suture is not tied

Now a Babcock or Duval clamp is used on the stapled edge of the PA to pull the PA towards the surgeon and a Lee bronchus clamp is placed at the base (Fig. 20A). The occluded Lee bronchus clamp is now looped with an unfolded gauze (raytec) and secured to the drape with a hemostat. The Babcock clamp is removed and the staple line is trimmed to transect the PA (Fig. 20B). The recipient and donor pulmonary arteries are trimmed to an appropriate length to prevent redundancy. It is very important step as a long pedicle can cause a twist in the anastomosis and cause PA obstruction. We often use 5–0 polypropylene 13 mm 3/8 circle taper point needle suture for anastomosis. This first stitch is taken backhand out-in on the recipient PA and in–out from donor PA with a rubber shod on the end. Then, the surgeon turns to the head end and takes all forehand bites. Once at the far end of the anastomosis, the surgeon turns towards the foot end and takes forehand bites of the anterior wall and stops midway. The surgeon then finishes off the anastomosis by bringing the other end forehand to meet midway on the anterior wall (Fig. 20C and Fig. 21). The PA is flushed with heparinized saline (5000u Heparin/500 cc NS). The two ends of the suture are left untied and fixed to the drape with a rubber shod.

**Fig. 22:**
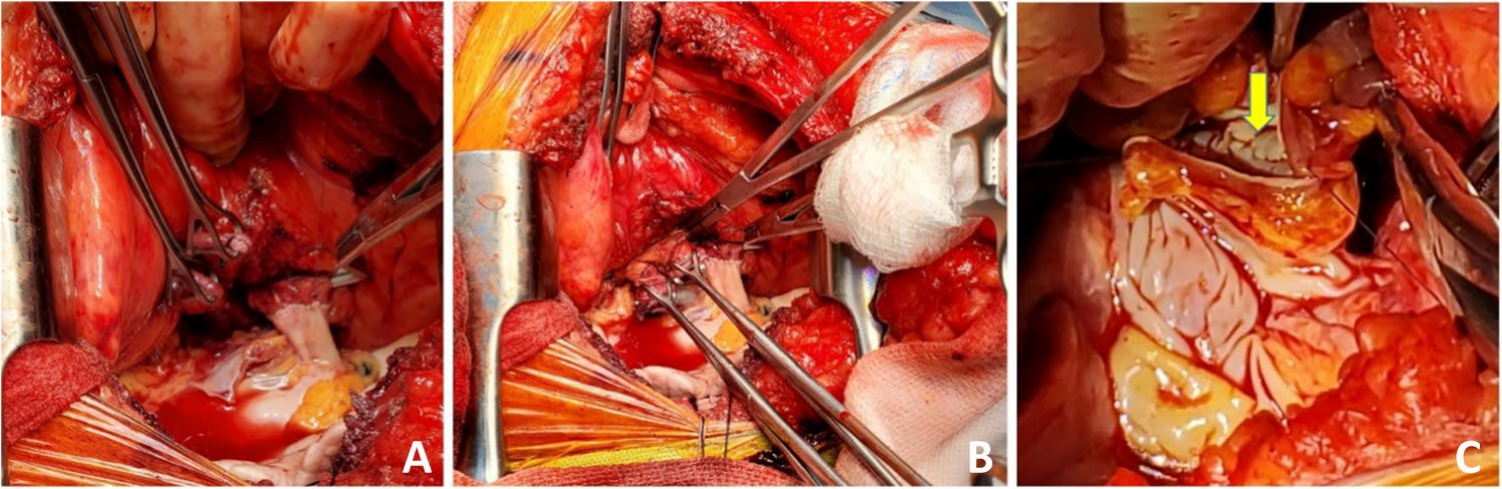
Set up for LA anastomosis. **A** The pulmonary veins being retracted by two Babcock clamps. **B** Satinsky clamp being placed at the base of the LA well beyond the bifurcation. **C** Completed posterior wall anastomosis of LA (yellow marker)

Two Babcock clamps or Duval clamps are used to hold the divided ends of the superior and inferior pulmonary veins to pull the left atrium (Fig. 22A). A Satinsky clamp is used to place it at the base of the left atrium. Care is taken to ensure it is placed beyond the drainage of the pulmonary veins (Fig. 22B). The occluded Satinsky clamp is looped with an unfolded gauze (raytec) and secured to the drape with a hemostat.

**Fig. 23:**
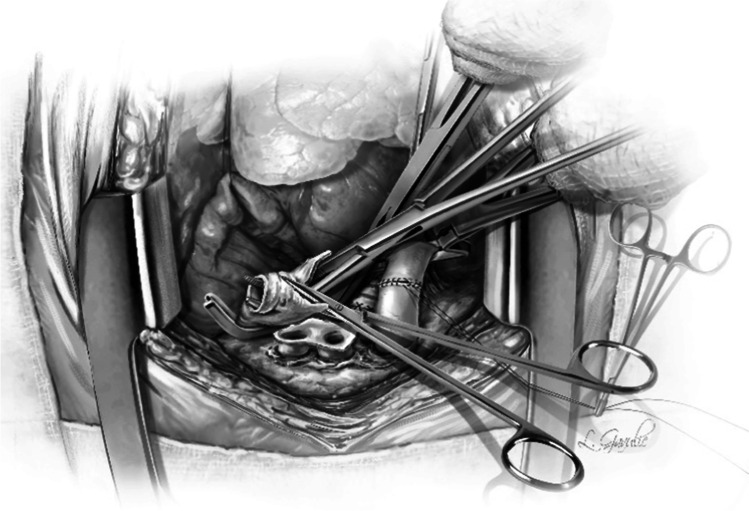
A right-angled clamp is passed through both veins and the LA is being opened

The clamps are removed and the pulmonary veins are opened. A right-angled clamp is passed from superior pulmonary vein and to the inferior and spread opened and cut open the bridge using Metzenbaum scissors (Fig. 23). The LA margins are fashioned (care must be taken to make sure that the posterior lip is not too small). A 4–0 polypropylene suture with 17 mm half circle taper point needle is used for this anastomosis. We perform it similar to the PA anastomosis with the first bite being backhand. The back wall is anastomosed first. It is very important to get endothelium to endothelium edges in an everting fashion (Fig. 22C, please also refer to figures in the right lung anastomosis later in the text).

**Fig. 24:**
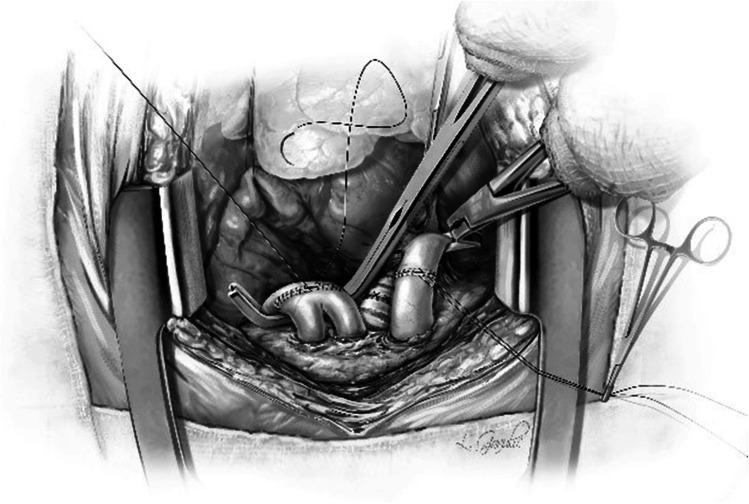
Completed LA anastomosis. Note both PA and LA sutures are not tied at this stage

One way to do this is by Imbrication of the edges of the donor LA. This can also be achieved by taking half thickness bites on donor LA and full thickness bites on recipient LA to achieve endothelium to endothelium apposition. Another technique is to hold on to the recipient LA with the forceps as the suture is tightened. This is clearly shown in the latter figure showing the right lung implantation (Fig. 34). Once the posterior wall is anastomosed, the anterior wall is approximated endothelium to endothelium by over and over everting sutures (Fig. 24). It is believed that this suture line, if improperly done (not achieving intima to intima apposition), has a risk for thrombus formation, post-operative stroke, or pulmonary vein thrombosis.

**Fig. 25:**
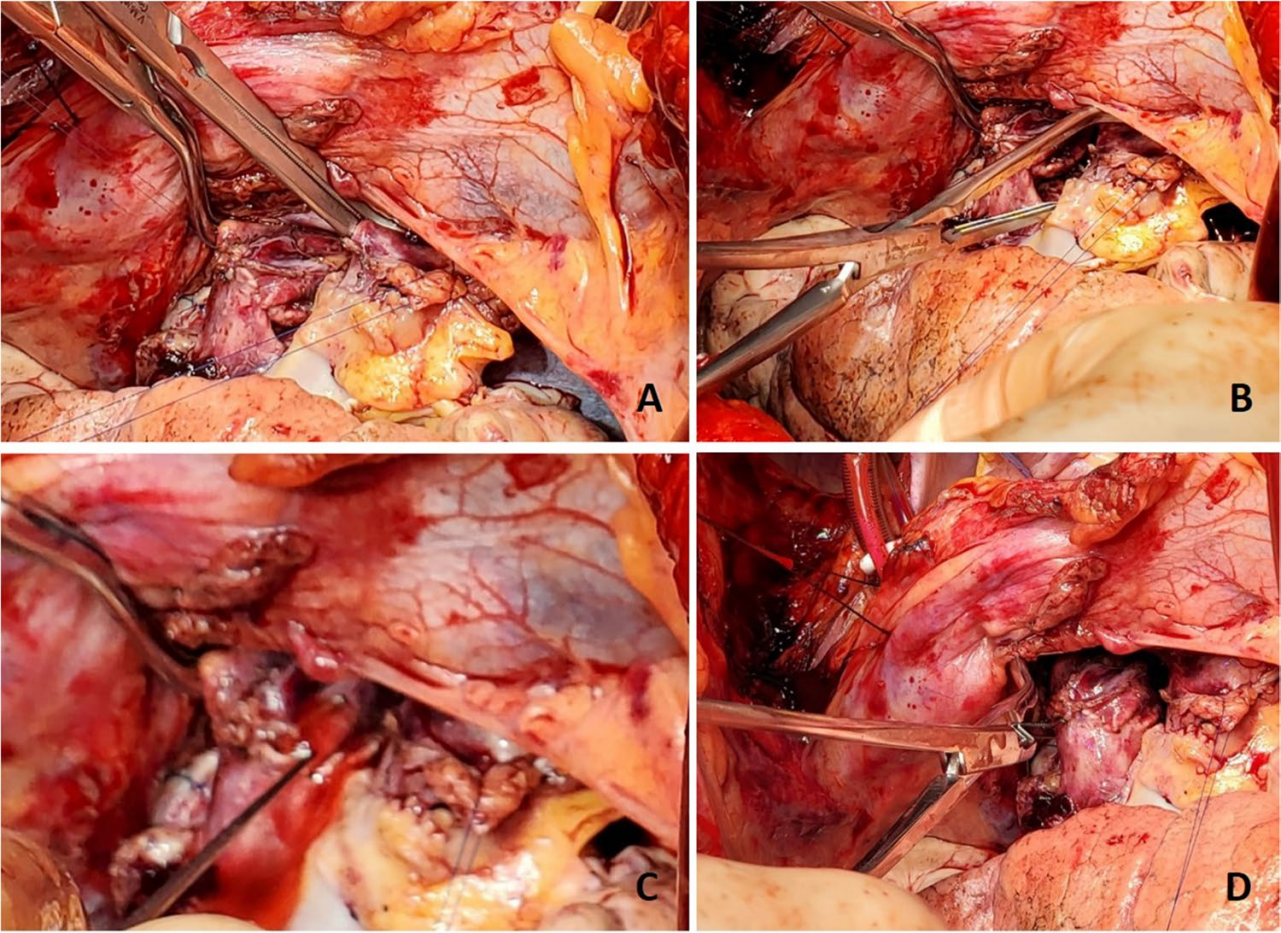
Showing the sequence of retrograde de-airing in a right lung. **A** Both clamps are on and both sutures are untied. **B** The Satinsky clamp removed from LA, keeping both ends of suture under tension. **C** The suture line of the PA is loosened using a nerve hook to drain the remaining Perfadex in the lung and also for retrograde de-airing. **D** Removal of the Lee bronchus clamp from the PA

When the LA anastomosis is completed, the sutures are left untied (Figs. 24 and 25A), and LA is flushed with heparinized saline (5000u Heparin/500 cc NS).

The FIO2 is reduced to 21% on the ventilator (similarly if on CPB, the FiO2 delivered to the oxygenator is reduced to 21%) and the de-airing maneuvers are performed, beginning in a retrograde direction. To accomplish this, the Satinsky clamp on the LA is released which flushes all the Perfadex solution in the lung to the PA while keeping the LA suture tight (Fig. 25B). Now without opening the PA clamp, the sutures are loosened to allow egress of retrograde flush out (Fig. 25C). Usually, it is the stored Perfadex from the lung, which is flushed. Then, an antegrade flush is done by releasing the Lee bronchus clamp from the PA slowly (Fig. 25D) and the sutures are tied. The LA suture is now tied.

The timer measuring warm implantation time is stopped after release of the LA clamp. If needed, any repair sutures are placed at this point. At this stage, often there is bleeding from the donor pericardial edges which can be managed by cauterizing the donor pericardial edge (this step also can be done prior to implanting). Now the left lung is inflated on room air to total lung capacity.

### Right lung implantation

**Fig. 26:**
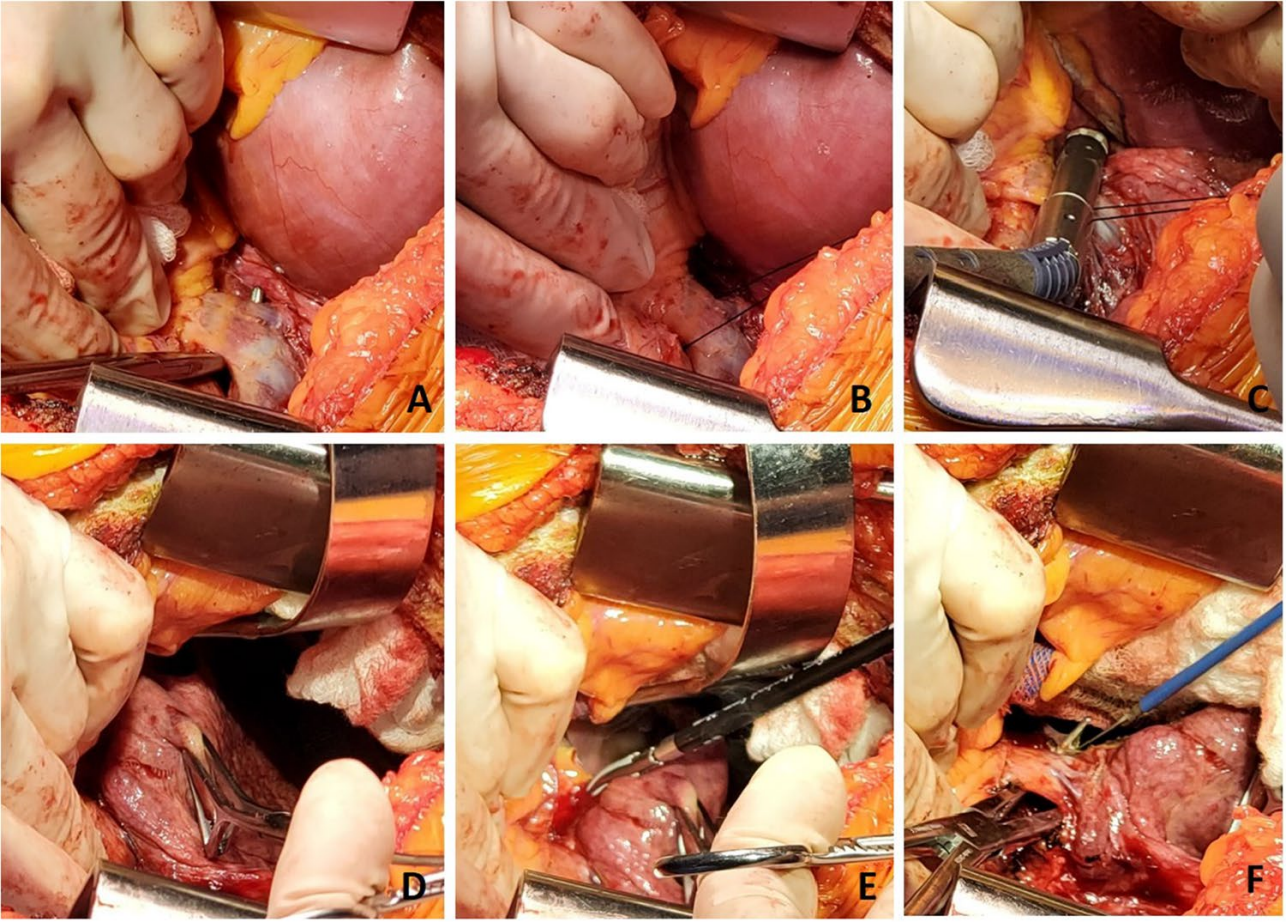
Dissection of right pulmonary veins. **A** Right-angled clamp loops the pulmonary vein. **B** Silk suture passed around. **C** Stapled across with Flex 45 stapler. **D** Duval clamp used to lift the inferior lobe. **E** The inferior pulmonary ligament is divided with LigaSure. **F** The inferior pulmonary vein is looped

**Fig. 27:**
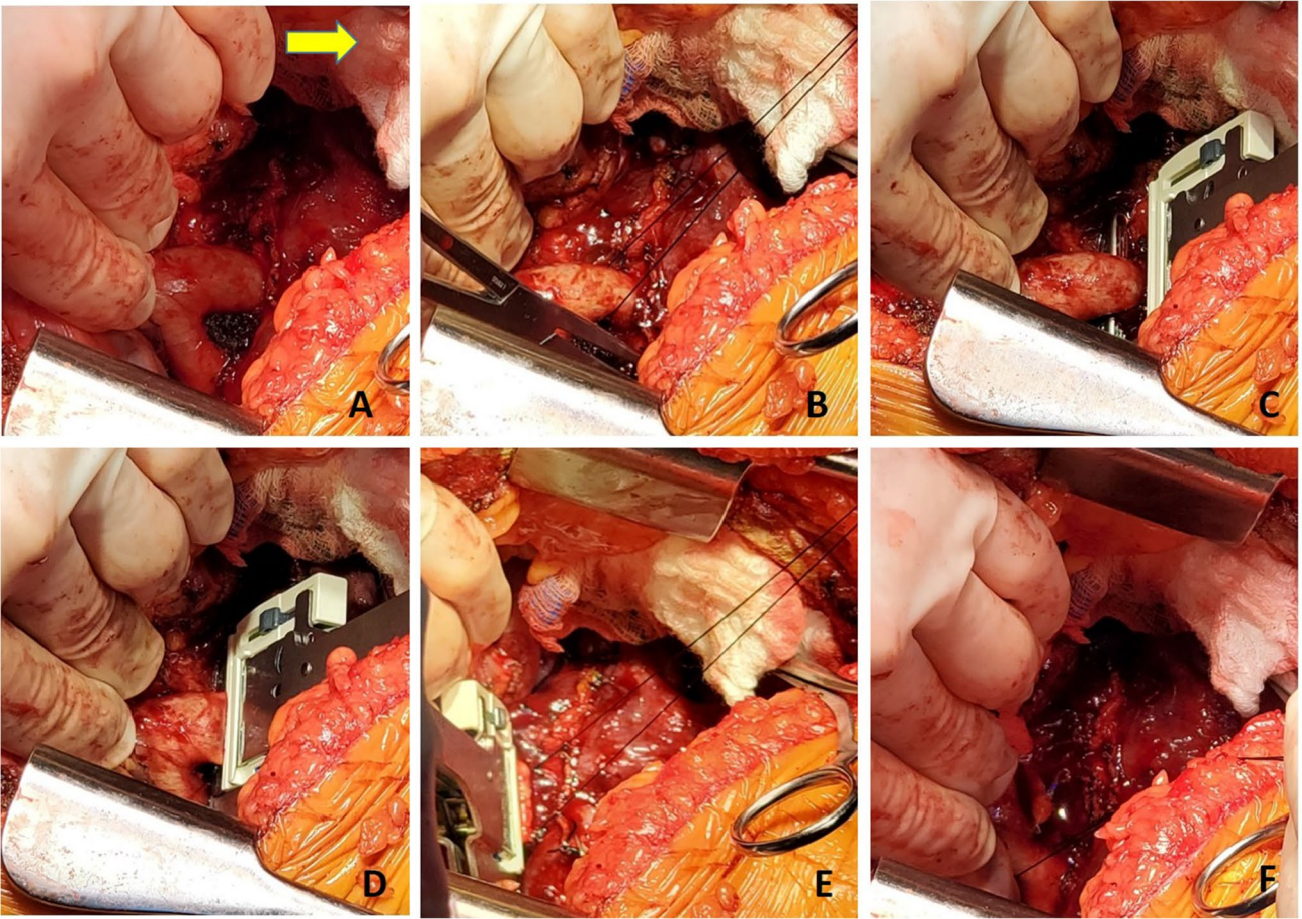
Dissection of the right pulmonary artery. **A** Note that the diaphragm has been pulled inferiorly with a silk stay suture with a rolled gauze (yellow marker) placed between the suture and the diaphragm. This silk suture is brought out through a separate incision and fixed with two hemostats pulmonary artery dissected showing the truncus intermedius and superior or truncus anterior branch. **B** The large intermedius branch looped with silk. **C** TX 30 vascular stapler (Ethicon Proximate TX Reloadable Linear Stapler) stapled distally. **D** Proximal stapling. **E** Divided between staple line. **F** Truncus anterior branch looped

**Fig. 28:**
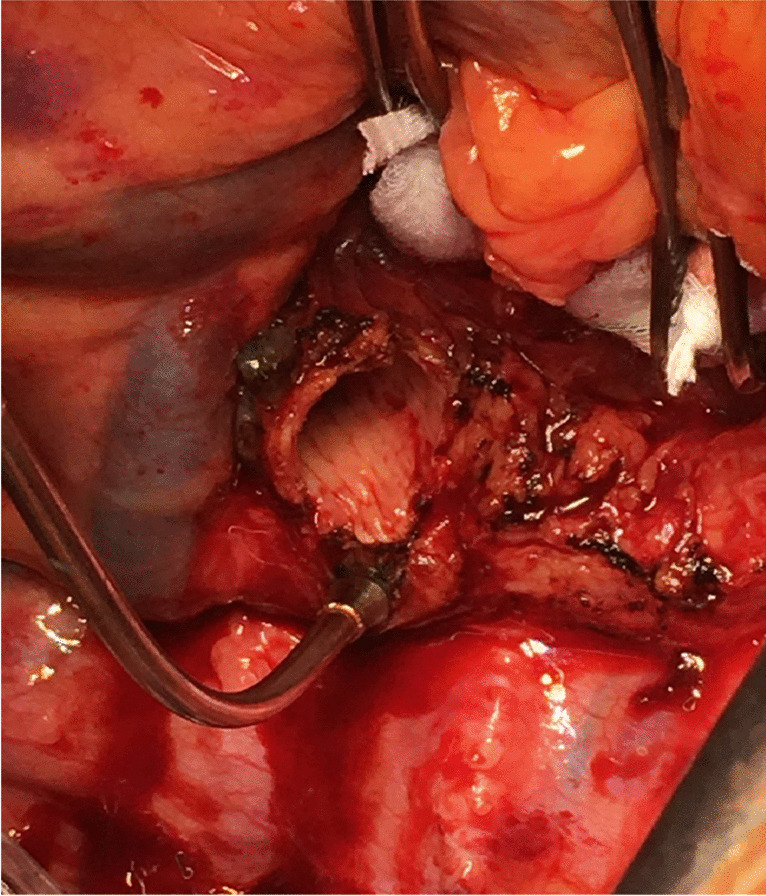
The dissected right bronchus stump is shown

A similar procedure is commenced on the opposite, right side. With the transplanted left lung being ventilated, single lung ventilation is started and the right lung is collapsed. Often at this stage, the saturations decrease as there is a ventilation/perfusion (V/Q) mismatch. Intermittently, the lung needs to be expanded to prevent further desaturation and the pneumonectomy is performed expeditiously. Pneumonectomy is performed in the same manner as described for the left lung. Hilar structures are dissected in the following order. The pulmonary veins first (Fig. 26) followed by PA (Fig. 27A–F) and finally the bronchus (Fig. 28) is divided with a No.15 Bard scalpel and LigaSure (Medtronic LigaSure™ Maryland Jaw) for the membranous portion. The pneumonectomy is completed.

The cuffs are developed. First, the bronchus is trimmed and care is taken not to de-vascularize the bronchus (Fig. 28). The PA stump is well mobilized from the surrounding tissue. Finally, the pulmonary veins are dissected intra-pericardially as a LA cuff. The deepest para-costal sutures are placed first.

Occasionally, the right hemi-diaphragm is markedly elevated. Often, a traction suture is placed and brought out through the location which can be used for chest drain and is pulled down and fixed with two hemostats (Fig. 27A).

**Fig. 29:**
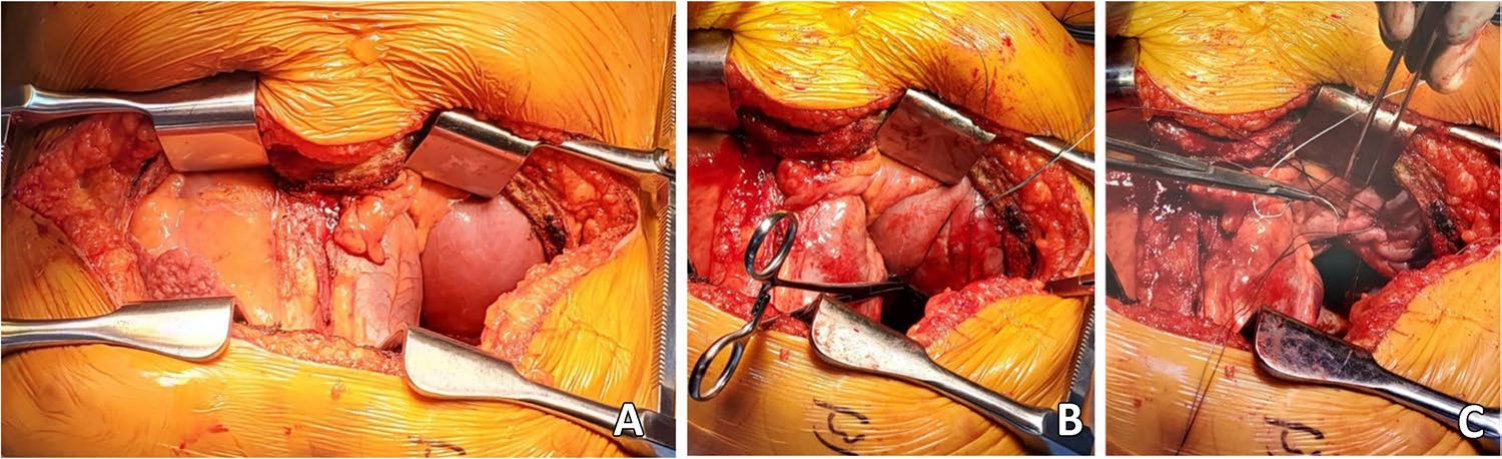
Right diaphragm plication after pneumonectomy. A view from head end. **A** Elevated diaphragm. **B** Serial pledgetted sutures taken. **C** Plication completed

If the diaphragm is very high, we do have a low threshold to formally plicate the right hemi-diaphragm with a series of horizontal mattress sutures using number 0 non-absorbable, braided suture. This is done before implanting the lung (Fig. 29).

**Fig. 30:**
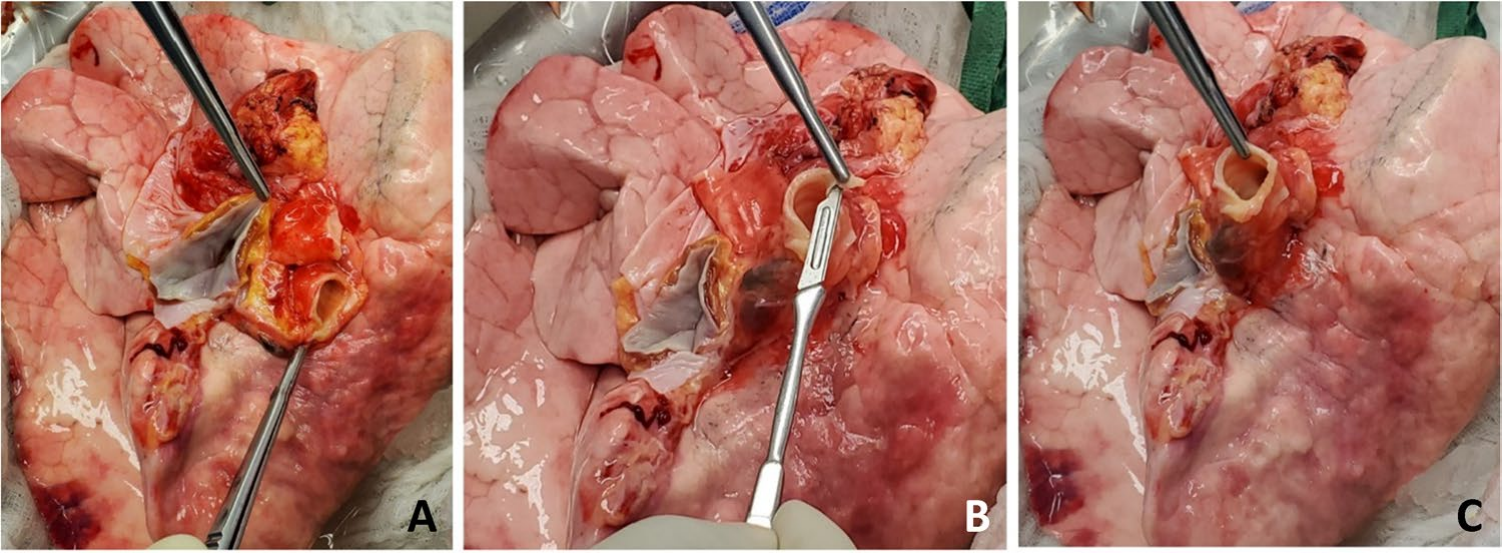
Back bench preparation of right lung. **A** Right LA cuff. **B** Donor bronchus fashioned with a no.15 blade. **C** Donor bronchus trimmed to 2 rings from the upper lobe take-off

The back bench preparation of donor lung is performed as explained for the left lung (Fig. 30).

**Fig. 31:**
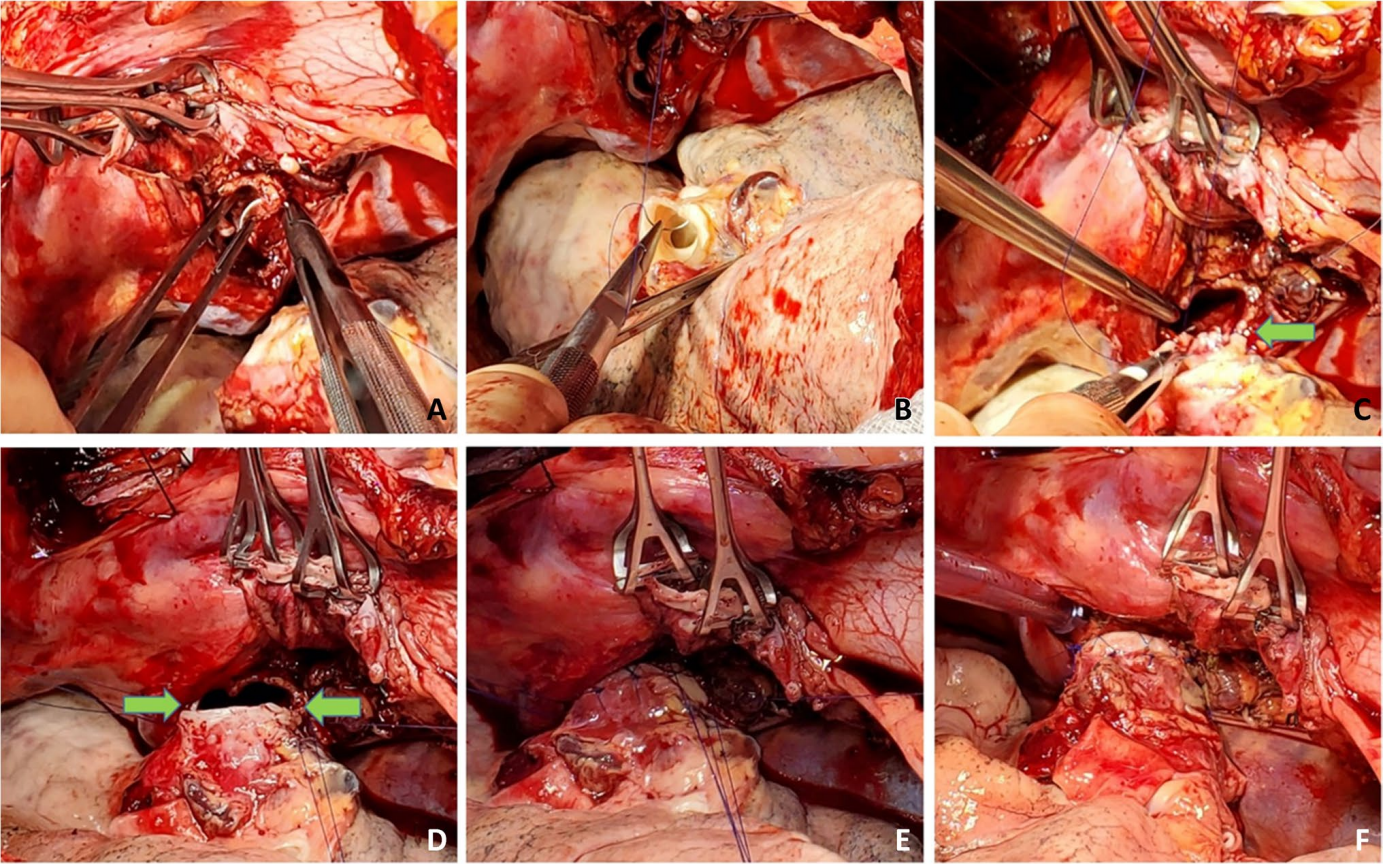
Right bronchus anastomosis. **A** First suture from outside-in on recipient at the membrano-cartilaginous junction. **B** The same suture taken from inside-out in the donor bronchus at corresponding end membrano-cartilaginous junction. C Completed membranous portion continuous suture (green marker). D Figure of “8” sutures started from either ends (green markers). E Sutures tied. F Completed anastomosis

**Fig. 32:**
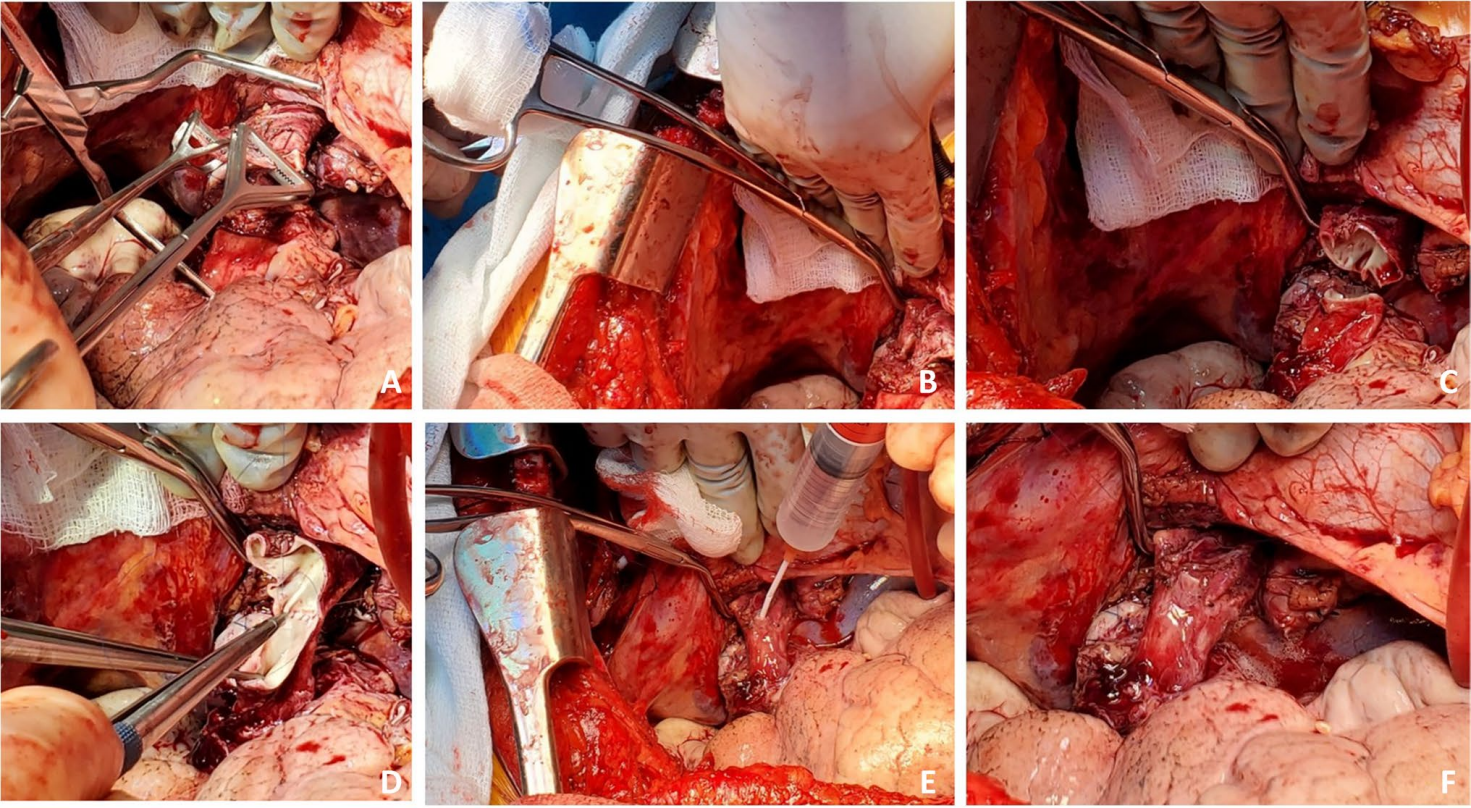
Right pulmonary artery anastomosis. **A** Duval clamp and Babcock forceps holding the stump of PA and a Lee bronchus clamp is being applied. **B** The clamp is being wrapped with an opened gauze and fixed to the drape. **C** Stapled ends have been cut open. **D** Anastomosis of PA. **E** Flush with heparinized saline. **F** Completed anastomosis with suture ends not tied

**Fig. 33:**
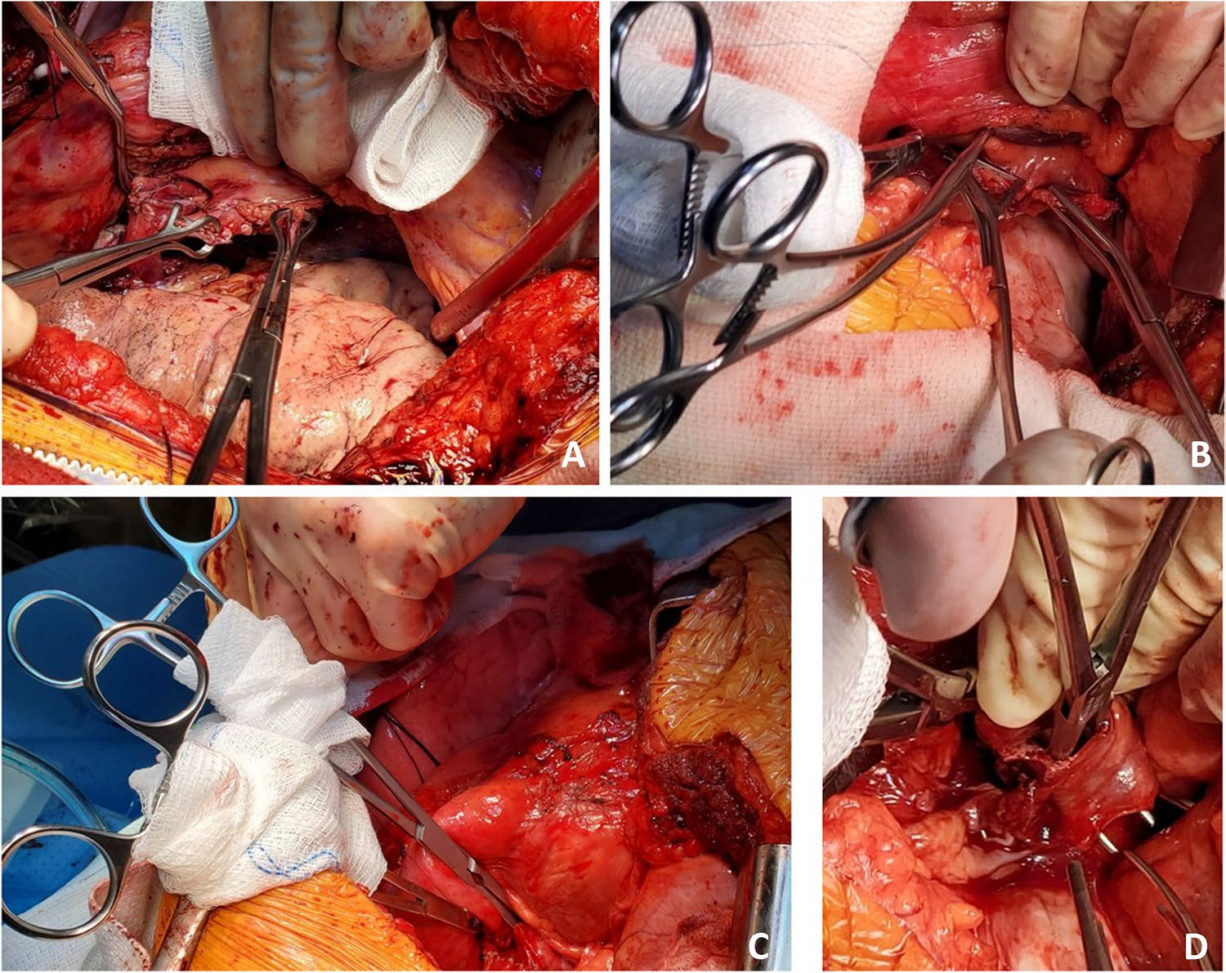
Left atrial anastomosis of the right lung. **A** The left atrial cuff being pulled by two Babcock forceps to the stapled pulmonary veins. Note the direction of the inferior Babcock forceps (along the stapled line which is antero-posterior). **B** Two Duval forceps being used to pull the pulmonary veins while the Satinsky clamp is being placed at the base. **C** The Satinsky clamp is wrapped with an opened gauze and fixed to the drape. Note that both the PA and LA clamps are fixed to the drape. **D** LA opened by passing right-angled clamp through both pulmonary veins

**Fig. 34:**
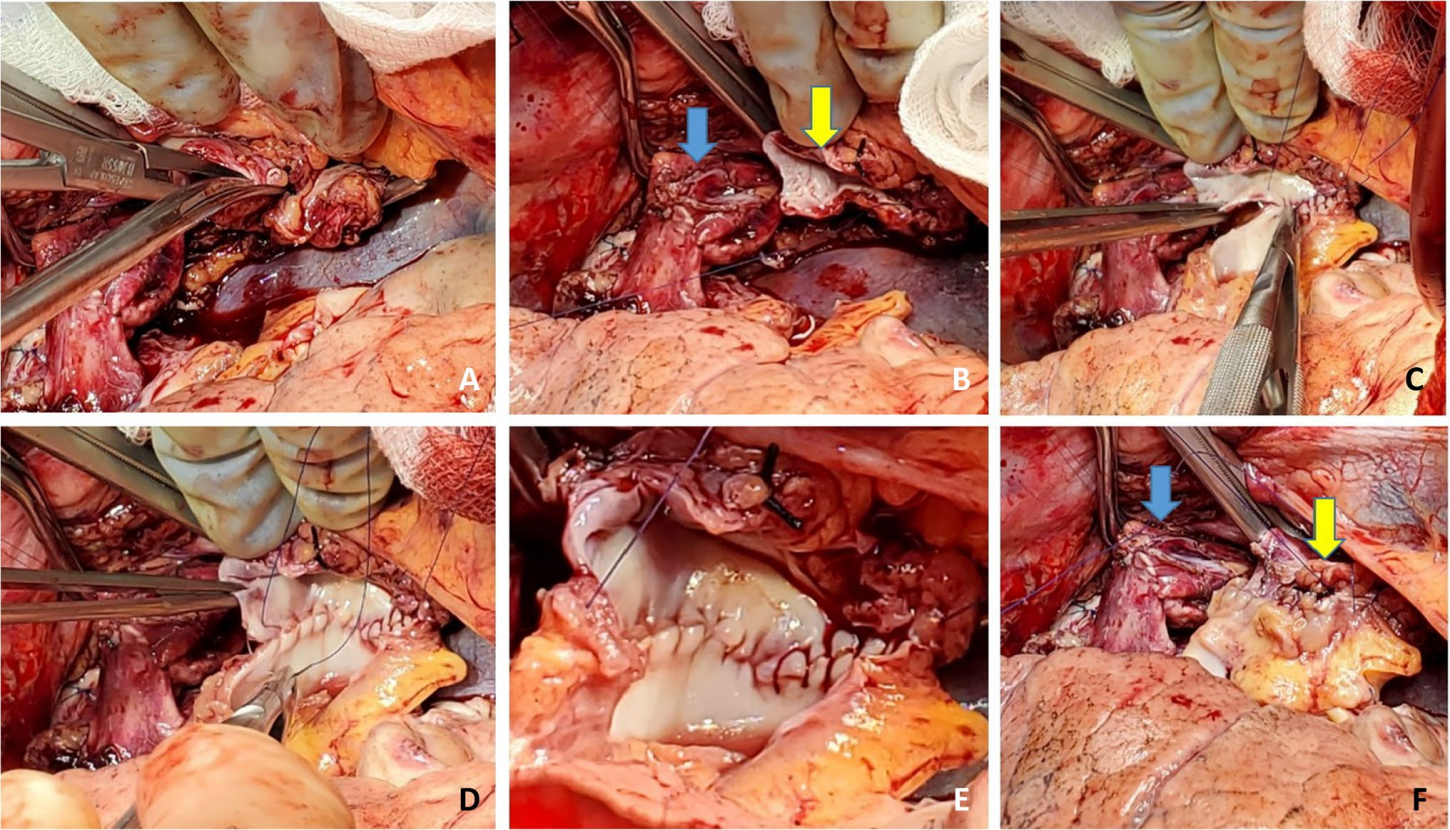
Left atrial anastomosis for the right lung. **A** The LA is cut opened. **B** LA donor cuff is fashioned (yellow marker). Note the PA anastomosis is completed (blue marker). **C** LA anastomosis started. **D** Note intima-to-intima anastomosis. **E** Completed posterior wall anastomosis with intimal apposition. **F** Completed PA (blue marker) and LA anastomosis (yellow marker)

The right lung is then implanted and all anastomosis are done identical to the technique described for the left lung (Figs. 31, 32, 33, and 34).

It should be noted here that it is better if the donor bronchus can be intussuscepted into the recipient (caution is taken on right side if recipient bronchus is intussuscepted into donor bronchus, as this may block the right upper bronchus).

Bronchoscopy should be done to confirm that the upper and lower lobes are widely patent.

FIO2 is decreased to 21%. De-airing maneuvers are accomplished (as described above in Fig. 25), first in a retrograde and then an antegrade fashion as the vascular clamps are removed and the vascular sutures are tied down. The right lung is inflated to total lung capacity on room air as well. Finally, both lungs are ventilated initially on room air and then the FIO2 is gradually increased.

### Closure

**Fig. 35:**
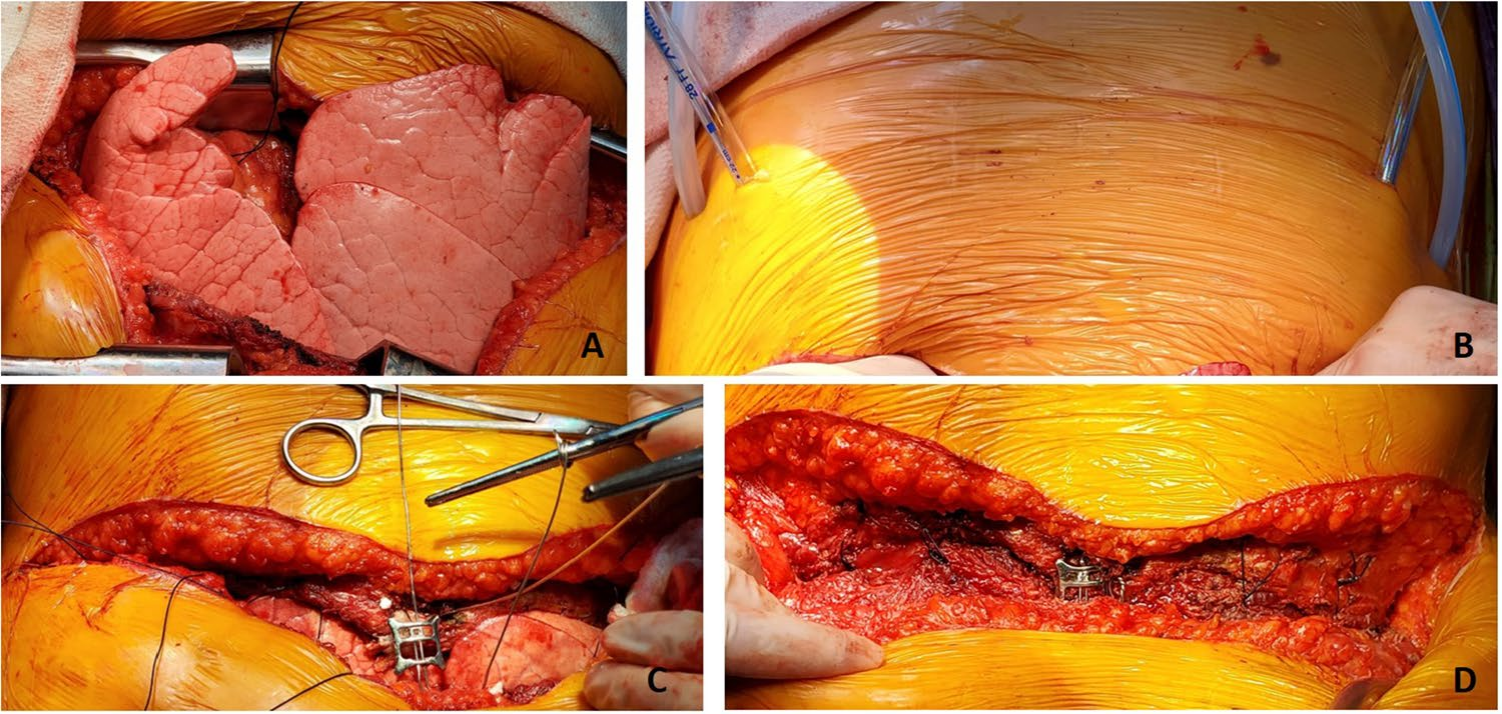
Sternal closure (all images are viewed from the head end). **A** Transplanted bilateral lungs. **B** Placement of drains bilaterally (medially straight drains and laterally Blake drains). **C** Multiple para-costal figure of “8” sutures with AcuTie sternal fixation of sternum. **D** Sutures tied and AcuTie and sternal wire tightened

The pericardium if opened is loosely re-approximated. Surgical hemostasis is secured (Fig. 35A). A 28 Fr anterior-apical straight chest tube is positioned along the hilum and a posterior-apical 24 Fr Blake drain are placed in each pleural space (Fig. 35B). Both drains are connected to − 20 cm suction.

**Fig. 36:**
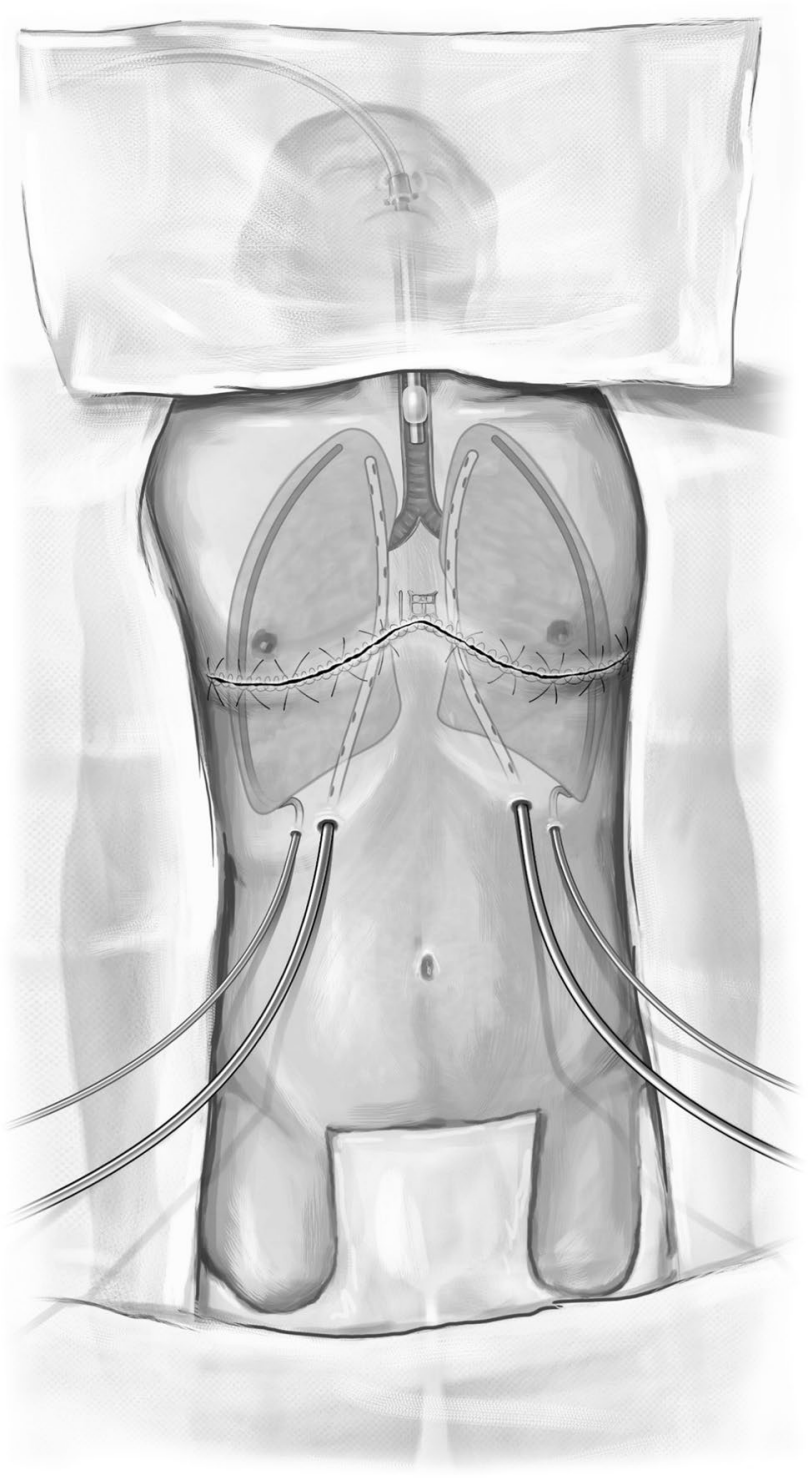
Chest closure and location of drains

In addition to the two previously placed para-costal sutures, an additional 4–6 No. 2 absorbable, synthetic, braided Polyglactin para-costal figure of eight sutures are placed (Fig. 35C). A single straight wire is placed in the sternum. A second wire is placed and an AcuTie Sternal Fixation device (Acute Innovations AcuTie II Sternal Fixation) is secured with the tightening and crimping tools (Fig. 35C and D). The first wire is tightened. Of note, recently we have started to utilize the ‘KLS Martin sternal plating system’ a multi-hole titanium plate with self-tapping cortical screws for our sternal re-approximation. At this stage, all members of the surgical team change gloves. The para-costal sutures are tied (it is often easier to tie the lateral most ties from the opposite side) while ventilation is held to prevent the lung from herniating between the rib space. When all of the para-costal sutures are tied, ventilation is resumed. The wound is irrigated out with warm saline. The pectoralis/serratus is closed with No.0 Polyglactin, subcutaneous fascia with No.2–0 Polyglactin, and subcuticular with No.3–0 absorbable synthetic monofilament suture. Prineo Dermabond (Ethicon DERMABOND® PRINEO® Skin Closure System) tape is applied. The completed chest closure and the location of drains is demonstrated in Fig. 36.

### Immediate post-operative

The double-lumen endotracheal tube is removed and replaced with a single-lumen endotracheal tube. Flexible fiber optic video bronchoscopy using an Olympus BF T180 flexible video bronchoscope is then performed by passing the video bronchoscope into the tracheal lumen through the endotracheal tube. Right and left main stem bronchi, right and left bronchial anastomoses are examined for patency, lobar obstruction, and anastomotic line. The bilateral upper lobes, right middle lobe, bilateral lower lobes, and bronchus intermedius are examined. Segmental and sub-segmental bronchi are examined, evaluating for any evidence of reperfusion pulmonary edema and bloody secretions. These are suctioned and the bronchoscope is withdrawn.

Once the patient’s hemodynamics are stabilized, we are able to transfer to the cardiothoracic surgery intensive care unit (CICU). Patients often require inotropic or vasopressor support (epinephrine or norepinephrine) initially, and intravenous infusion of Propofol and Tacrolimus are also frequently used. Patients may benefit from inhaled nitric oxide, particularly if there was pre-existing moderate-severe PAH or to facilitate off bypass implantation when PAH or hypoxia are noted on single lung ventilation. If the patient was on pre-operative ECMO support, we continue the same intraoperatively and wean it in the ICU once the patient is stable.

### If CPB is used

**Fig. 37:**
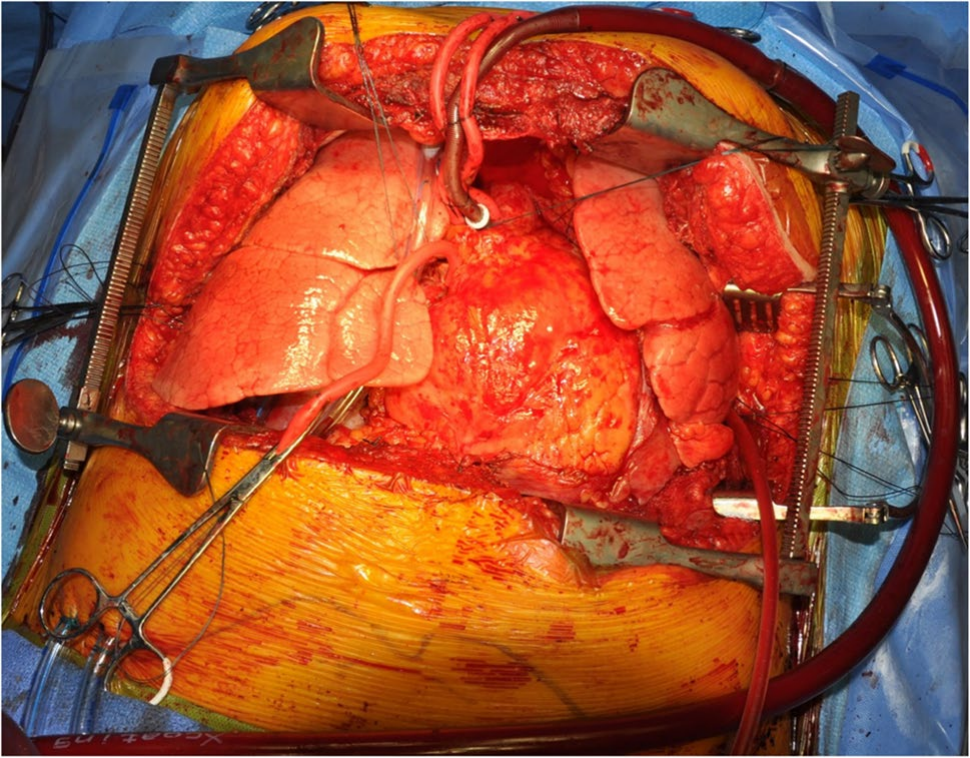
Bilateral LT on CPB

We often use the epi-aortic ultrasound to identify any concerning plaque in the ascending aorta. Systemic heparin is administered. The aorta is cannulated de-aired and connected to the bypass circuit and secured (Fig. 37). A two staged (cavo-atrial) venous cannula is passed through the right atrium (RA) and full CPB is established once activated clotting time (ACT) levels are greater than 420 s.

Occasionally, we prefer to use femoral cannulation, particularly if bypass is needed for single lung transplants.

It is important to note that after the anastomosis of the lung, the FIO2 is dropped to 21% on CPB and de-airing maneuvers are performed as described above in retrograde followed by antegrade fashion as the vascular clamps are removed and the sutures are tied.

Patients undergoing LT with the use of CPB may develop coagulopathy and are at increased risk for post-operative bleeding and blood product transfusion. Massive transfusion can be detrimental to newly transplanted lungs. Also, early post-CPB right ventricular function can be compromised. In these cases, we have a low threshold to leave the chest open, pack the cavity meticulously with long roller gauze packing, and close the skin only. Once stable, we return to the OR for delayed closure, usually 24–48 h later.

## Conclusion

LT is the only effective therapy for end-stage lung disease. Here, we describe our techniques for donor organ preparation and implantation. This method has been used for nearly 900 implants with excellent results and low perioperative mortality (less than 2% in the most recent era). We use EVLP to evaluate marginal or otherwise unacceptable donors. Our preferred approach for double LT is to perform the procedure sequentially without the use of CPB. When this is not tolerated either due to hemodynamic instability or hypoxia, we have a low threshold to use CPB, although this increases bleeding risk due to the need for systemic anticoagulation and associated coagulopathy. Of the 863 isolated (excluding the 24 multi organ transplants), we have used CPB in 286 patients (33%). Pre-operative ECMO support was used in 24 patients (22 had Veno-venous ECMO and 2 patients had Veno-arterial ECMO). While a successful technical implantation lays the foundation for a good outcome, multi-disciplinary pre-operative management, post-operative and long-term management is essential to ensure excellent long-term survival of these patients. In addition, successful LT program is a sum total of teamwork of transplant surgeons, transplant pulmonologists, infectious disease specialists, transplant nursing, physical therapists, respiratory therapists, social workers, psychologists, social workers, transplant coordinators, EVLP team, and many other teams making it one of the most challenging solid organ transplant programs.

## References

[1] Toronto Lung Transplant Group (1986). Unilateral lung transplantation for pulmonary fibrosis. N Engl J Med.

[2] Patterson GA, Cooper JD, Goldman B (1988). Technique of successful clinical double-lung transplantation. Ann Thorac Surg..

[3] Pasque MK, Cooper JD, Kaiser LR, Haydock DA, Triantafillou A, Trulock EP (1990). Improved technique for bilateral lung transplantation: rationale and initial clinical experience. Ann Thorac Surg.

[4] Meyers BF, Sundaresan RS, Guthrie T, Cooper JD, Patterson GA (1999). Bilateral sequential lung transplantation without sternal division eliminates post transplantation sternal complications. J Thorac and Cardiovasc surg.

[5] Lonchyna VA (1999). Single lung transplantation. Operative Tech Thorac Cardiovasc Surg.

[6] Davis RD (2007). Bilateral sequential lung transplantation. Operative Tech Thorac Cardiovasc Surg.

[7] Puri V, Patterson GA (2008). Adult lung transplantation: technical considerations. Semin Thorac Cardiovasc Surg.

[8] Boasquevisque CH, Yildirim E, Waddel TK (2009). Surgical techniques: lung transplant and lung volume reduction. Proc Am Thorac Soc.

[9] Aigner C, Klepetko W (2012). Bilateral lung transplantation. Operative Tech Thorac Cardiovasc Surg.

[10] Daneshmand MA, Lin SS, Haney JC, Hartwig MG. Bilateral sequential lung transplantation. Oper Tech Thorac Cardiovasc Surg. 2014;19:138–51.

[11] Sharma A, Peltz M, Wait MA (2020). The conduct of thoracic organ procurement. Asian Cardiovasc Thorac Ann..

[12] Subramaniam K, Rio JMD, Wilkey BJ (2020). Anesthetic management of lung transplantation: results from a multicenter, cross-sectional survey by the society for advancement of transplant anesthesia. Clin Transplant.

[13] Tomasi R, Betz D, Schlager S (2018). Intraoperative anesthetic management of lung transplantation: center-specific practices and geographic and centers size differences. J Cardiothorac Vasc Anesth.

[14] Nicoara A, Anderson-Dam J (2017). Anesthesia for lung transplantation. Anesthesiol Clin.

[15] Módolo NSP, Módolo MP, Marton MA, et al. Intravenous versus inhalation anaesthesia for one-lung ventilation. Cochrane Database Syst Rev. 2013. 10.1002/14651858.CD006313. 10.1002/14651858.CD006313.pub3 PMC6464685 23846831

